# Medical‐financial partnerships for improving financial and medical outcomes for lower‐income Americans: A systematic review

**DOI:** 10.1002/cl2.70008

**Published:** 2024-12-06

**Authors:** Julie Birkenmaier, Brandy R. Maynard, Harly M. Blumhagen, Hannah Shanks

**Affiliations:** ^1^ Saint Louis University School of Social Work St. Louis Missouri USA

**Keywords:** financial outcomes, healthcare interventions, health outcomes, medical‐financial partnerships, social determinants of health

## Abstract

**Background:**

Poverty is considered one of the social determinants of health (i.e., a range of social and environmental conditions that affect health and well‐being) because of its association with significant health problems. In recent years, healthcare settings have emerged as focal points for poverty interventions with direct health implications. Medical institutions are increasingly implementing financial partnerships to provide interventions targeted at improving the financial well‐being of patients with the dual objective of boosting appointment attendance rates and alleviating financial burdens on patients. While medical‐financial partnerships (MFPs) appear to be growing in popularity, it is unclear if these interventions positively impact financial and/or health outcomes.

**Objectives:**

The purpose of this review is to inform policy and practice relevant to MFPs by analyzing and synthesizing empirical evidence related to their health and financial outcomes. The primary objectives of this review is to answer the following research questions: (1) What is the extent and quality of MFP intervention research? (2) What are the effects on financial outcomes of financial services embedded within healthcare settings? (3) What are the effects on health‐related outcomes of financial services embedded within healthcare settings?

**Search Methods:**

We conducted a comprehensive search for published and gray literature from September to December 2023. We searched for and retrieved published studies from Google, Google Scholar, and 10 Electronic databases. We also searched five relevant websites and two trial registries for registered studies. We harvested from the reference lists of included studies and conducted forward citation searching using Google Scholar. Lastly, we contacted the first authors of the four included studies and requested information about unpublished studies, studies in progress, and published studies potentially missed in the other search activities.

**Selection Criteria:**

Studies eligible for this review met the following criteria. First, studies must have used a prospective randomized controlled trial or quasi‐experimental (QED) research design with parallel cohorts. Second, studies must have involved an intervention that provides financial services on‐site within a healthcare setting. Third, the studies must have measured a financial outcome. Fourth, to meet the criteria for on‐site financial services, interventions must have included at least one of the following: (1) financial education, counseling or coaching, (2) credit counseling, or (3) the provision of services that assist patients to access financial products or services, such as free tax preparation services, or (4) services to increase income, such as screening for public benefits and assistance with the application process, as well as employment services (e.g., assistance with resume writing and job interviewing skills). Health‐related outcomes were extracted and analyzed, but were not required for eligibility.

**Data Collection and Analysis:**

Searches were saved in the reference management software EndNote2, and duplicates were removed and uploaded to Rayyan. Four reviewers then completed title and abstract screening on 66,807 entries in Rayyan. Three reviewers independently reviewed the 26 articles that were moved forward for full‐text screening. A fourth reviewer reviewed discrepancies and made the final decision to include or exclude. Four studies that satisfied the inclusion criteria were retained for data extraction using a standardized extraction form. Because the included studies did not measure and report sufficient data to calculate effect sizes for similar outcomes, quantitative synthesis was not possible. Effect sizes were calculated when possible, and study outcomes were described.

**Main Results:**

Of the four unique studies included in this review, two were randomized control trials and two were QEDs. Three of the four studies were conducted in pediatric settings. Two of the studies focused on tax preparation only as their financial intervention, both offering a VITA tax clinic on‐site in the healthcare clinic setting. One study featured financial coaching, which included a bundle of services such as one‐on‐one case management, budgeting and goal setting, and the fourth study provided financial counseling over two sessions delivered remotely. All four studies reported at least one financial outcome, and two studies reported at least one health‐related outcome. The evidence on the health and financial impacts of MFPs is limited due to the small number of included studies and lack of authors reporting data to calculate effect sizes. Results indicate small and nonsignificant effects of MFPs on financial outcomes reported, and some author‐reported positive statistically significant effects on attending appointments and adhering to vaccination schedules. The risk of bias assessment indicated important methodological weaknesses across included studies.

**Authors' Conclusions:**

Although MFPs are becoming popular and have the potential to improve financial and health outcomes, there is an overall lack of evidence about whether MFPs are meeting their goals. Very few studies met inclusion criteria, and those that did were generally low quality, and therefore, we were unable to draw any conclusions regarding the intervention effects. Given the nascent nature of the research, the high level of enthusiasm for MFPS seems to be outpacing the evidence about their effectiveness on important outcomes. We advocate that healthcare settings invest first in rigorous research on pilot MFPs and disseminate their findings widely before making a determination about taking them to scale in practice and/or move ahead to integrate them into healthcare policy.

## 1 PLAIN LANGUAGE SUMMARY

### 1.1 Limited evidence on the health and financial outcomes of medical‐financial partnerships (MFPs)

MFPs lack sufficient evidence to conclusively demonstrate their effectiveness in improving financial and health outcomes within healthcare settings.

#### 1.1.1 What is this review about?

Poverty and financial stress have been linked to negative impacts on health, and more attention has been given to addressing financial strain as a means of improving health. As healthcare settings increasingly become a focus for poverty interventions to improve health outcomes, MFPs, or collaborations between medical facilities and financial service providers, have gained popularity. MFPs are interventions that include financial education, counseling or coaching, credit counseling or other financial services delivered in a healthcare setting. This review examined both financial and health outcomes.

#### 1.1.2 What is the aim of this review?

The aim of this review was to understand the quality and extent of research on MFP interventions in the United States, as well as the effects of embedding financial services within healthcare settings on financial and health‐related outcomes. This review summarizes evidence from four studies, including two randomized and two quasi‐experimental studies.

#### 1.1.3 What are the main findings of this review?

This review included studies that assessed the effects of MFPs on financial and health outcomes. A total of four studies were identified, with all four studies reporting at least one financial outcome and two studies reporting at least one health outcome. Most studies were conducted in a pediatric care setting. Two studies provided tax preparation services and the other two provided financial coaching or financial counseling. All studies had some important methodological weaknesses.

#### 1.1.4 How has this intervention worked?

Results indicate small and non‐significant effects of MFPs on financial outcomes and one study reported positive statistically significant effects on attending appointments and adhering to vaccination schedules. Due to the small number of studies, the small sample sizes, and methodological weaknesses of included studies, there is a lack of evidence of the effects of MFPs on financial and health outcomes.

#### 1.1.5 What do the findings of this review mean?

The findings of this review suggest that while MFPs may hold promise for improving financial and health outcomes, there is currently insufficient robust evidence to support their effectiveness. This lack of clear evidence calls for rigorous research of MFPs in healthcare settings using RCTs and standardized outcomes, and studies that evaluate components of MFPs for their effectiveness on the outcomes desired.

#### 1.1.6 How up‐to‐date is this review?

The review authors searched for studies up to September 2023. This Campbell Systematic Review was published in November, 2024.

## 2 BACKGROUND

### 2.1 The problem, condition or issue

Despite social safety net programs such as Social Security and the Earned Income Tax Credit (EITC) that provide income to the lowest income Americans, poverty continues to be a widespread problem in the United States. According to the US Census Bureau (2022), about 12% of Americans, or 38 million people, have household incomes below the U.S. poverty line. Poverty is considered one of the social determinants of health (i.e., a broad range of social and environmental conditions that affect health and well‐being) because of its association with significant health problems (Francis et al., 2018). Poverty affects adult and child physical health due to its impact on access to safe housing, healthy foods, employment and educational opportunities, healthcare services, clean air and water, and a safe neighborhood environment (US Department of Health and Human Services, n.d.). Poverty is a special concern for minoritized populations, as poverty disproportionately impacts racial and ethnic minorities (Board of Governors of the Federal Reserve, 2021). States with higher levels of services that ameliorate poverty have significantly better health outcomes (Bradley et al., 2016).

Financial stress and strain is often experienced for those living below and just above the federal poverty level ($31,200 annually for a family of four in 2023). Financial strain is experienced when unexpected expenses occur, such as a health bill or car repair, even for households that regularly meet daily and monthly expenses. About half of U.S. households have less emergency savings than the amount needed to cover expenses for 3 months in the absence of income, as recommended by financial planners (FINRA, 2022). In 2022, only 63% of adults could cover a hypothetical unexpected $400 expense using cash, savings or a credit card paid off at the next statement (Board of Governors of the Federal Reserve System, 2023). Households without savings often take on unmanageable debt, which can result in debt collection proceedings. Hassani and McKernen (2018) estimate that 32% of adults, or 71 million people, have debt in collections. The lack of savings is also critical for older adults; half of all adults nearing retirement have no personal retirement funds (King, 2022).

Financial stress and strain negatively affects health and well‐being through several pathways. First, chronic financial and other types of stress lead to the acceleration of normal aging through the shortening of telomeres and increases in allostatic load, together called “weathering.” These processes lead to premature aging and earlier onset of unfavorable physical health conditions (Geronimus, 2023). Second, financial stress interferes with regular medical care due to foregone care as a result of lack of affordability of care or other poverty‐related access challenges. Preventive medical care can decrease in priority for families facing financial stress, which in turn can result in conditions becoming more acute and requiring more intervention (Schickedanz et al., 2023). Racial and economic discrimination faced by people of color, people with disabilities and other groups can compound financial stress (Geronimus, 2023). Children in low‐income households whose parents are under financial stress may suffer harm to their cognitive and social/emotional development due to harsh, inconsistent, and/or detached parenting resulting from the stress (National Academies of Sciences, Engineering, and Medicine, 2019).

Poverty interventions are often housed within the social services sector (e.g., Birkenmaier et al., 2022). However, healthcare settings are increasingly a topic of interest for poverty interventions that have health implications (Rosenberg & Sude, 2024). Primary and tertiary healthcare settings that target and/or serve primarily lower‐income and communities of color are trusted healthcare settings that serve populations that could benefit from services that lower financial stress (South et al., 2022). Primary healthcare settings provide low‐ or no‐cost preventive and primary healthcare, while tertiary healthcare settings provide hospital‐based care. These settings often function as the first point of contact in the healthcare system because referrals are not needed to access care; therefore, they can provide a gateway to other services. Primary healthcare providers are focused on the comprehensive and interrelated aspects of physical, mental, and social health, and are thus particularly interested in the home environment and stressors from patients. Tertiary healthcare settings with hospital clinics and wellness centers can also incorporate financial services to reduce financial stress (Bell et al., 2020).

Health practitioners are also focused on improving health outcomes, particularly through finding ways to promote keeping appointments focused on preventative care (Rosenberg & Sude, 2024). Primary healthcare clinics that serve low‐income families have high no‐show rates for appointments – estimates range between 25% and 50% of all appointments (Samuels et al., 2015). A relatively recent arrangement called “Medical Financial Partnerships” (MFPs) often have the dual goal of improving “show” rates for appointments and improving financial stress (Schickedanz et al., 2023).

### 2.2 The intervention

MFPs, a relatively new and innovative intervention, are currently being piloted within healthcare settings that serve lower‐income and Medicaid‐insured patients. These partnerships aim to directly address issues of patient financial stress and poverty (Liu et al., 2021), and promote adherence to healthcare appointments (Schickedanz et al., 2023). Typically initiated by health delivery systems, such as clinics, hospitals, or broader health networks, MFPs involve collaboration with financial service organizations to offer services that enhance both community finances and health. Distinguishing themselves from previous models, MFPs integrate financial stability efforts into the healthcare provision, attempting to create a synergistic effect on community wellness (Bell et al., 2020; Hole et al., 2017). The purpose of this service delivery model is to foster a robust connection between healthcare and financial services, offering a spectrum of support from specific interventions such as free tax preparation to more comprehensive services (Marcil et al., 2021). The goal of MFP services is to provide a platform to enhance patient financial stability for patients through a variety of offerings, including but not limited to, free tax preparation, enrollment in savings accounts, employment assistance and workforce development, financial coaching, and/or assistance with applying for benefit programs. MFPs serve a wide demographic, including hospital and clinic patients, their employees, and the wider community. MFPs provide financial services both onsite and in the broader community through formal organizational partnerships (Marcil et al., 2021).

Several key factors are driving the development and offering of MFPs. The idea that financial stress is inherently a healthcare issue is gaining traction. Prominent professional and government entities, including the Academy of Pediatrics and the American Academy of Family Physicians, advocate for healthcare providers to address the social determinants of health, which includes financial stress (American Academy of Family Physicians, 2022; Gitterman et al., 2016). Additionally, the National Association of Community Health Centers and the Centers for Medicare and Medicaid Services have developed tools for screening for the social determinants of health (Center for Medicare and Medicaid Services, n.d.; National Association of Community Health Centers Inc. (NACHC), 2022). Academic research further supports the significance of such screening tools in recognizing the social determinants of health, including poverty (Gruss et al., 2021; LaForge et al., 2018; O'Gurek & Henke, 2018). News coverage of MFPs is also driving interest (Huang, 2023).

Moreover, primary healthcare settings have historically incorporated non‐financial social resources. Initiatives addressing necessities such as food security (Adams et al., 2017; Palakshappa et al., 2017), as well as behavioral health and legal services (Beck et al., 2021) have become integral components of patient care. Notably, some healthcare settings have incorporated Medical‐Legal Partnerships, which embed legal services addressing legal barriers to financial wellness, such as benefit or disability appeals, workplace discrimination, and family medical leave (Marcil et al., 2021). Furthermore, qualitative research findings suggest patients are interested in services integration, such as financial coaching and free tax preparation clinics (Liu et al., 2021). Individual studies suggest that the integration of financial services within healthcare settings could lead to improved financial outcomes, such as reduced debt and higher credit scores, and better health outcomes through reduced financial stress, increased adherence to healthcare appointments, and greater utilization of preventive healthcare services (Dalembert et al., 2021; Schickedanz et al., 2023).

### 2.3 How the intervention might work

MFPs are pioneering approaches that operate on‐ and off‐site of healthcare facilities through partnership arrangements. This review distinguishes on‐site MFP interventions from conventional social work services in healthcare settings, and will focus particularly on those interventions delivered on‐site and through tele‐consultations with financial service providers operating under a healthcare provider's service model. While social workers in healthcare settings typically refer patients to external resources such as financial counseling and coaching and emergency nutrition supplements (Browne, 2019), MFPs that are colocated are distinctly different from traditional social services, and provide unique features. First, colocation simplifies access to services for patients to access services, whether in conjunction with their healthcare visits or as standalone services. Secondly, these services are integrated into the healthcare framework, and emphasize a holistic approach to patient care (Marcil et al., 2021). On‐site MFP interventions can offer a suite of financial services, including financial counseling, financial coaching, tax preparation services, credit counseling, debt consolidation and forgiveness, and employment assistance (Bell et al., 2020). Patients can access these services within the physical space of healthcare settings (Dalembert et al., 2021; San Francisco General Hospital, n.d.), or via tele‐consultation.

Community partnerships are integral to these colocated services. Toward that goal, the American Academy of Pediatrics has created a model for developing and sustaining effective community partnerships. This framework aids healthcare providers in identifying and aligning with partners to ensure and sustain patient benefits while efficiently managing staff resources (American Academy of Pediatrics, 2021). In practice, various healthcare staff members inform patients about the available financial services and encourage their utilization.

Across the MFP models currently implemented, financial coaching and tax preparation services are most prominent (Dalembert et al., 2021; Marcil et al., 2021). Financial coaching has evolved into a standardized, evidence‐based method that empowers low‐income families toward financial well‐being (Hall et al., 2022; Modestino et al., 2019). Financial coaches assist clients to set and pursue their financial goals, complemented by financial education, and strategies to enhance budgeting, savings, and credit. Financial coaches work one‐on‐one or in groups, and use motivational interviewing and financial planning techniques, as well as assist to apply for public benefits (Collins et al., 2021). Demonstrated outcomes of financial coaching include increased financial knowledge and confidence, reduced stress and debt, and improvements in income, savings, and credit scores (Collins et al., 2021; Silva et al., 2022). Some MFP models include financial counseling (Sadigh et al., 2022). Financial counseling, in contrast, is a service that provides assistance on a particular issue, such as debt management, and often includes financial education.

In MFP models that include financial coaching, coaches maintain communication with clients between medical visits, and focus on financial stress and progress toward client‐specific goals. The aim is to foster supportive, trusting, and nonjudgmental relationships that bolsters patient financial capability and adherence to healthcare appointments by aligning services with the patient's financial objectives. This approach seeks to build trust with the healthcare team, encouraging engagement with both healthcare and financial services (Schickedanz et al., 2023).

Free tax preparation within the MFP framework addresses another vital need (Bell et al., 2020). Filing for federal income taxes, particularly for low‐income families eligible for significant refunds through credits such as the EITC and the Child Tax Credit (CTC), represents a crucial annual financial opportunity. Yet, an estimated 20% of those eligible fail to claim these benefits (Internal Revenue Service, 2022), and Black and Latinx children in families that are disproportionately affected (Bruenig & Williams, 2021). Free tax preparation assistance through programs such as the Volunteer Income Tax Assistance (VITA) program and Tax Counseling for the Elderly (TCE) is available for those earning $60,000 or less annually, people with disabilities, and those with limited English proficiency (US Department of Housing and Urban Development, n.d.), potentially offsetting the $1.75 billion earned by the for‐profit tax industry annually (Weinstein & Patten, 2016).

Additionally, MFPs provide a variety of other services, such as employment assistance, diaper distribution, assistance with food insecurity, Child Development Accounts, housing assistance, savings programs, benefits enrollment, FAFSA assistance, pre‐k enrollment, and medical bill negotiation, enhancing the holistic support offered to patients (Marcil et al., 2021).

### 2.4 Why it is important to do this review

This review has significant relevance for both health and financial policy. First, health policy is increasingly focused on the crucial role of the social determinants of health, including financial factors, in influencing health outcomes (World Health Organization, 2023). By addressing the financial goals and needs of patients, which can enhance health outcomes, policy may shift due to the reduced healthcare costs that arise from increased preventative healthcare delivery, such as vaccinations and health screenings. The findings of studies in this domain could inform health policy, particularly in the context of primary healthcare. Furthermore, the existing body of evidence provides little support for the idea that financial services, such as financial coaching and free‐tax preparation clinics, could increase their impact when integrated into familiar and trusted environments, such as health clinics (Alexander et al., 2022). The effectiveness of embedding financial services within such settings could inform policies related to these services. For instance, the demonstrated success of MFPs might motivate policymakers to integrate these services more comprehensively into health systems and establish funding mechanisms for financial services.

Despite recent interest in MFPs, research remains fragmented, and to date, no systematic review of the MFP intervention focusing on their financial and health outcomes has been published. While one scoping review (Parry et al., 2021) has explored financial interventions within healthcare systems aimed at addressing poverty in high‐income countries, it did not adhere to systematic review methodologies. Bell et al. (2020) undertook a systematic review of various MFP models and described different organizational approaches, without focusing on financial and/or health outcomes. Thus, no systematic reviews, to date, have examined MFP financial and/or health‐related outcomes.

The research in this area is emerging, with scholarly discussions starting around 2017. Our preliminary search yielded several quantitative studies (Marcil et al., 2018; Marcil & Thakrar, 2022a; Markowitz et al., 2022; Schickedanz et al., 2023) and qualitative studies (Bennett et al., 2021; Jaganath et al., 2018; Liu et al., 2021; Marcil & Thakrar 2022b; Quinn et al., 2018), but the evidence is dispersed across disciplines. Given the interdisciplinary nature of this research and the novelty of these interventions, conducting a systematic review is crucial for assessing the components of the intervention implemented, the outcomes measured, and the strength of the evidence to effectively inform policy and practice.

This study addresses knowledge gaps in several areas. While individual studies on certain MFP interventions have observed positive outcomes, such as financial coaching (Theodos et al., 2015), and tax‐time saving interventions (Roll et al., 2020), recent systematic reviews on other interventions offered within MFPs, such as matched savings accounts (Birkenmaier et al., 2022), Child Development Accounts (Birkenmaier et al., 2022), tax‐time saving interventions (Birkenmaier et al., 2023), and on financial services interventions broadly, including financial coaching (Birkenmaier et al., 2022) have reported mixed or modest effects on savings amounts or were inconclusive. Other reviews, including systematic reviews, have found that financial education, counseling, and coaching has little or no effects on subsequent behavior, including short‐term saving (Birkenmaier et al., 2022; Fernandes et al., 2014; Kaiser & Menkhoff, 2020; Miller et al., 2015). Thus, further systematic reviews of financial services interventions are necessary.

### 2.5 Objectives

The purpose of this review is to inform policy and practice relevant to MFPs by analyzing and synthesizing empirical evidence related to their health and financial outcomes. The primary objectives of this review is to answer the following research questions using formal research studies:

1. What is the extent and quality of MFP intervention research?
2. What are the effects on financial outcomes of financial services embedded within healthcare settings?
3. What are the effects on health‐related outcomes of financial services embedded within healthcare settings?

## 3 METHODS

The conduct and reporting of this review followed the Methodological Expectations of Campbell Collaboration Intervention Reviews (MECCIR). The *protocol* for this review was published in Campbell Systematic Reviews (Birkenmaier et al., 2023).

### 3.1 Criteria for considering studies for this review

#### 3.1.1 Types of studies

To mitigate threats to internal validity, studies must have used a prospective randomized controlled trial (RCT) or quasi‐experimental (QED) research design with parallel cohorts. Comparison groups could have included those on a waitlist, attention control, or standard care. Comparison or control groups could have received their needed healthcare, but could not have financial services within the healthcare setting aside from passive referral. It is possible that comparison/control group members could have received financial services outside of the healthcare setting. Studies using single‐group pre–post test design, or single subject design, or historical comparisons were excluded.

#### 3.1.2 Types of participants and settings

All populations were included. Participant dyads, such as caregiver‐patient and caregiver‐infant were also eligible for inclusion. All U.S. healthcare clinical settings where primary, secondary, or tertiary healthcare services are provided were eligible to be included.

#### 3.1.3 Types of interventions

MFPs require intentional collaboration between medical facilities and/or staff and financial service providers. Studies eligible for this review had financial services embedded within the medical facility and could have been delivered in person or remotely (e.g., video‐conference). Though many medical settings make referrals to local financial assistance resources, this practice alone does not qualify as an MFP and was understood as “treatment as usual” for the purposes of this review.

To meet the criteria for on‐site financial services, interventions must have included at least one of the following: (1) financial education, counseling or coaching, (2) credit counseling, or (3) the provision of services that assist patients to access financial products or services, such as free tax preparation services, matched savings accounts or special child savings accounts to be used for education purposes, or (4) services to increase income, such as screening for public benefits and assistance with the application process, as well as employment services (e.g., assistance with resume writing and job interviewing skills). Financial services could be provided by any group, organization, or agency, whether non‐profit or for‐profit, whose primary role is to provide such services. The services are provided to the patients who use the healthcare setting for any duration of services. Financial services that were provided for the sole purpose of affording healthcare or services designed to help lower the cost of medication were excluded from the review.

#### 3.1.4 Types of outcome measures

##### Primary outcomes

To meet the criteria for outcome measurement, studies must have measured and reported a financial outcome as a primary outcome. These outcomes could include financial knowledge and attitude, tax filing status, tax refund and receipt and amount, debt, savings, and credit scores. Studies may also report a health care outcome, such as visit adherence, immunization schedule adherence, and other health indicators.

##### Secondary outcomes

Secondary outcomes for inclusion consisted of:

(1) Knowledge about tax filing and tax credits (e.g., EITC, CTC) (Marcil et al., 2018).
(2) Outcome related to healthcare usage (e.g., feel more connected to healthcare providers) (Marcil et al., 2018).
(3) Outcomes related to child patients of participating parents. These outcomes can include education goals for children whose parents participate in a child special savings account program.
(4) Outcomes related to employment services. These outcomes can include outcomes related to resume assistance, job search assistance, employment application and enrollment assistance, and income from paid employment.
(5) Outcomes related to income security unrelated to employment, such as accessing public benefits.
(6) Outcomes related to microfinance and emergency finance.
(7) Adverse or unintended outcomes reported in primary studies.

#### 3.1.5 Duration of follow‐up

Interventions of any length or duration were eligible for inclusion, from single‐session services such as tax preparation to multi‐session financial coaching offerings.

#### 3.1.6 Types of settings

All healthcare settings were eligible as long as financial services were offered on‐site. Eligible healthcare settings included (1) pediatric clinics (primary or specialty), (2) federally‐funded healthcare clinic (FQHC) (primary or specialty, all ages), (3) hospital or ambulatory sites affiliated with a hospital (off main‐site), (4) primary health clinics (non‐pediatric), (5) older adult setting (assisted living, memory care, rehabilitation facilities), or (6) other healthcare settings not covered in the previous categories.

Studies where financial services were delivered outside of the healthcare setting were excluded. Studies conducted outside the United States were excluded due to the U.S. healthcare system being unique among high‐income countries in not providing universal health care, and intervention effectiveness may be unique because of the healthcare system.

#### 3.1.7 Other criteria

Studies must have been published in English, which was due to being consistent with focusing on studies conducted in the United States as well as due to limitations in resources to translate studies published in other languages. Although we did not anticipate finding studies on this intervention before 2017, we did not restrict inclusion of studies based on the date of when studies were conducted or published. We included both published and unpublished studies.

### 3.2 Search methods for identification of studies

We conducted a comprehensive search for published and gray literature informed by Campbell's guide for searching for studies (Kugley et al., 2017). We searched multiple electronic databases, gray literature sources, reference lists of reviews and relevant studies, trial registries and relevant websites and contacted authors.

#### 3.2.1 Electronic searches

Following the procedures outlined in the protocol, we searched for and retrieved studies through a comprehensive search that included Google, Google Scholar, and 10 Electronic databases (Platform): ABI/INFORM (ProQuest); Academic Search Complete (EBSCOhost), Business Source Premier (EBSCOhost); ProQuest Dissertations & Theses Global; EconLit (EBSCOhost); PubMed; APA PsychINFO (Ovid); SCOPUS (Elsevier): Social Science Research Network (Elsevier): Social Sciences Citation Index (Clarivate). Database searches were conducted in September 2023. See Supporting Information S1: Appendix A for the full search strategies used in each database. Searches were not limited with regard to publication date.

#### 3.2.2 Searching other resources

In addition to the database searches, we searched for studies on websites and study registries. Websites searched included the Centers for Disease Control and Prevention, the World Bank, OECD, the Global Partnership for Financial Inclusion, and the Alliance for Financial Inclusion, and Social Interventions Research and Evaluation Network (SIREN) by the University of San Francisco California. We also searched Clinicaltrials.gov, and the International Clinical Trials Registry Platform (WHO; https://who.int/ictrp/network/en/) for registered studies.

The reference lists from included studies were harvested for potential studies. We conducted forward citation searching using Google Scholar to search for studies citing the included studies. We contacted the first authors of the included studies and requested information about unpublished studies, studies in progress, and published studies potentially missed in the other search activities.

### 3.3 Data collection and analysis

#### 3.3.1 Selection of studies

Searches were saved in the reference management software EndNote21. Results were saved in two separate EndNote libraries to accommodate the volume of initial returns. Two members of the review team removed duplicates in the respective libraries using EndNote duplicate detection software. The cleaned data from each library were then imported into Rayyan, a cloud based application for conducting systematic reviews (Ouzzani et al., 2016). Once combined, we used the auto‐resolve feature within Rayyan to conduct an initial duplicate screen of entries with 97% similarity or more. All potential duplicates remaining after auto‐resolve were divided up between two team members and resolved manually.

Initial title and abstract screening on the remaining unique entries was divided between four reviewers and completed in December 2023. A total of 26 were moved forward for full‐text screening. One reviewer gathered full‐text on all retained studies.

Three reviewers independently conducted a blinded full text review of each study for eligibility. All reviewers included notes on any decisions to exclude. Discrepancies were reviewed by a fourth reviewer, who made the final decision to include or exclude. After full review, studies that met the inclusion criteria were retained for data extraction and analysis. Of the four included studies, three of them had registered protocols with clinicaltrials.gov that we used along with the studies for data extraction.

#### 3.3.2 Data extraction and management

The research team utilized a data extraction form to standardize data capture and coding (see Supporting Information S2: Appendix B). The types of data extracted included aspects of the intervention delivered, methods used, results, and outcomes. The team conducted a joint data extraction review on one study as a pilot to review and revise extraction methods. Three reviewers then independently extracted data from the studies. The full team evaluated the results of the extraction to assess for discrepancies or errors before finalizing the data. Discrepancies were reviewed and resolved through discussion. The final codes were saved in Excel.

#### 3.3.3 Assessment of risk of bias in included studies

Four review authors independently conducted a risk of bias assessment on each study using the Risk of Bias in Non‐randomized Studies of Interventions (ROBINS‐I) (Sterne et al., 2016) for studies that used QEDs and the Cochrane Collaboration's Revised risk‐of‐bias tool for randomized trials (RoB 2) for studies that used randomized control trial methods (Higgins et al., 2019). These tools are routinely used for assessing risk of bias in Campbell and Cochrane systematic reviews.

Using the ROBINS‐I to assess the non‐randomized studies, the review authors assessed the risk of bias for each of the following seven domains: due to confounding, in selection of participants into the study, in classification of interventions, due to deviations from intended interventions, due to missing data, in measurement of outcomes, and in selection of the reported result. Following the structure of the ROBINS‐I, each study was coded as “low,” “moderate,” “serious,” “critical” risk of bias or “no information” on each of the domains, and overall.

For the RoB 2, review authors assessed risk of bias for each of the following five domains: arising from the randomization process, due to deviations from intended interventions, due to missing outcome data, in measurement of the outcome, and in selection of the reported result. Each study was coded as “low,” “some concerns,” or “high” risk of bias on each of the domains, and overall. Following independent coding by four review authors, discrepancies were resolved through consensus. Three of the four studies had registered protocols with the National Institute of Health, National Library of Medicine (at clinicaltrials.gov) that assisted in the risk of bias assessment (Sadigh et al., 2022 (NCT0425707); Schickedanz et al., 2023 (NCT03736590), and Marcil & Thakrar, 2022a, 2022b (NCT04135469)).

#### 3.3.4 Measures of treatment effect

We calculated effect sizes for all outcomes of interest where study authors provided sufficient data. As anticipated, study authors reported both continuous and dichotomous outcome measures. For continuous outcomes, we calculated the magnitude of effect using the standardized mean difference effect size with Hedges' *g* correction. For dichotomous outcomes, we calculated the odds ratio. All effect sizes were calculated with the Practical Meta‐Analysis Effect Size Calculator (Wilson, n.d.).

#### 3.3.5 Unit of analysis issues

None of the included studies used a clustering or crossover design. For studies that reported multiple outcome measurement time points, we calculated and reported effect sizes for each time point. If there would have been a sufficient number of studies reporting the same outcome at similar time points, we would have pooled similar time points together; however, we did not have a sufficient number to quantitatively synthesize, and thus we reported effects for all reported time points in narrative and tabular format.

#### 3.3.6 Criteria for determination of independent findings

We assessed each of the included studies to ensure they were each reporting outcomes for unique study samples. If we found multiple reports of the same study, we used all of the information from each report, but counted it as only one study. Because we did not pool effects across studies, in cases where study authors reported different outcome measures for conceptually similar outcomes, we calculated effect sizes for each outcome and reported each effect size in narrative and tabular formats.

#### 3.3.7 Dealing with missing data

All studies that met inclusion criteria were included, regardless of whether there was missing data. For studies where there was not sufficient data to calculate an effect size, we contacted study authors to request the data.

#### 3.3.8 Assessment of heterogeneity

We planned to conduct a test of homogeneity (*Q* test) to compare the observed variance to what would be expected from sampling error, and the *I* ^2^ statistic to describe the percentage of total variation across studies due to heterogeneity rather than chance. We also planned to construct a forest plot displaying study‐level mean effect sizes and 95% confidence intervals for the included studies to provide opportunity for visual analysis of the precision of the estimated effect sizes, detection of studies with extreme effects, and information regarding the heterogeneity of studies. Due to the small number of included studies and the variation across studies regarding the outcomes reported, we did not conduct meta‐analysis and instead provided a narrative description of the included studies, thus we did not examine effect size heterogeneity.

#### 3.3.9 Assessment of reporting biases

Due to the small number of studies, we did not have a sufficient number of studies to assess publication bias as planned using a funnel plot. We did assess reporting bias by comparing reported outcomes in the included studies to the studies' protocols for three of the included studies that had published protocols and reported our findings narratively.

#### 3.3.10 Data synthesis

We had planned to conduct separate meta‐analysis to pool studies for each outcome construct using random effects statistical models. Due to the small number of included studies that measured a variety of different outcomes, we did not have a sufficient number of effect sizes measuring the same outcome construct to synthesize effects across studies. Instead, we calculated effect sizes for each outcome for which we had sufficient data to calculate effect sizes and reported these in narrative and tabular format.

#### 3.3.11 Subgroup analysis and investigation of heterogeneity

Moderating variables of interest included study design (RCT and QED), publication status (published or unpublished), dosage and duration of intervention (continuous variable), age (children or adults), and type of intervention (financial coaching, tax filing assistance, or other). We had anticipated that most of these studies would be conducted with low income groups and a mix of racial/ethnic groups, thus we did not anticipate conducting moderator analysis related to SES/income or race of participants. Due to the small number of included studies, however, subgroup analysis was not possible.

#### 3.3.12 Sensitivity analysis

We did not conduct any sensitivity analyses.

#### 3.3.13 Treatment of qualitative research

We did not include qualitative research in this review.

#### 3.3.14 Summary of findings and assessment of the certainty of the evidence

We did not plan to include a summary of findings and assessment of the certainty of the evidence.

## 4 RESULTS

### 4.1 Description of studies

#### 4.1.1 Results of the search

**Figure 1:**
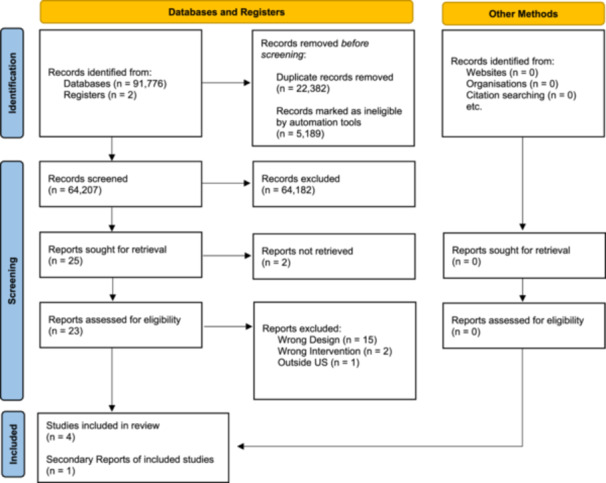
PRISMA flow diagram: search process and results.

The results of the search and selection process are illustrated in Figure 1. Electronic searches of bibliographic databases and searches of other sources yielded a total of 91,778 hits. Of the 91,778 hits, 91,776 were from database searching, and two were registered protocols. As part of the automatic deduplication process available in Rayyan outlined above, 5189 articles were removed as duplicates automatically. An additional 22,382 reports were manually identified as duplicates and removed. After deduplication, 64,207 titles and abstracts were screened for relevance and 64,182 were deemed inappropriate. The full text of the remaining 25 potential studies were reviewed and screened for eligibility by four independent coders. We excluded 20 reports that were deemed ineligible and included five reports that met the inclusion criteria. Of the five, one was a correction to an eligible report. This resulted in four unique studies (using unique samples) published in five reports that were included in this review. Published protocols were available for three of the four studies (Marcil & Thakrar, 2022a, 2022b; Sadigh et al., 2022; Schickedanz et al., 2023). We conducted forward citation searching and reference list searching on the included studies. None of the additional entries generated by these searches met the inclusion criteria for full‐text review. No new studies were located from websites, clinical trial registries, or from included authors. See the references of included studies for a list of reports that reported on the included studies.

#### 4.1.2 Included studies

A summary of the four included studies is presented in Table 1, and study‐by‐study details are provided in Table 2. Two of the four included studies were randomized control trials (Sadigh et al., 2022; Schickedanz et al., 2023) and two were QEDs (Marcil & Thakrar, 2022a, 2022b; Markowitz et al., 2022). The analytic sample sizes ranged from 40 to 126 participants.

**Table 1:** Summary of Characteristics of Included Studies (*k* = 4).

**Report Type**		**Funding Mechanism**	
Journal article	4	Private	4
**Predominant Sex**		**Other Service Provided**	
Female	4	Diaper distribution	1
**Method of Assignment to Condition**		**Participants**	
RCT	2	Adults	3
QED	2	Adult/child dyad	1
**Control/Comparison Group Received**		**MFP Setting**	
Treatment as usual	3	Pediatric clinic	3
Nothing or wait list	1	Adult specialty care	1
**Financial Services Delivery Platform**		**Predominant Race**	
Remote (option or standard)	2	African American	3
In person	2	Not specified	1
**Financial Program Source**		**Providers of Financial Program**	
Government/public	2	Nonprofit volunteers	2
For‐profit company	1	For‐profit staff	1
Nonprofit organization	1	Students	1
**Financial Services Training**		**Number of Sessions**	
IRS Tax preparation	2	1	2
Financial coaching/motivational interviewing	1	2	1
Not specified	1	Not specified	1
**Length of Treatment**		**Length of Each Session**	
4–6 months	1	20–40 min	1
3 months	1	1 h	1
Not specified	2	Not specified	2
**Adult Mean Age**		**Frequency of contact**	
30.5–35	2	Patient determined	1
41.5	1	Once	2
Not specified	1	Two visits	1
**Analytic Sample Size**		**Income**	
40	1	Low Income	1
81	2	Low‐moderate	1
186	1	Not specified	2
**Financial Services Provided**			
Assistance in applying or obtaining benefits and resources^a^	2	Goal setting^a^	1
Budgeting^a^	1	One‐on‐one case management^a^	1
Community resources screening and referral^a^	2	Public benefits screening and referral^a^	2
Financial coaching^a^	1	Tax preparation	2
Financial counseling	1		
**Outcomes**			
**Health**		**Financial**	
Care non‐adherence	1	Financial worry	1
Mental health quality of life	1	Income	1
Physical quality of life	1	Material hardship	1
Vaccination adherence for child	1	Receipt of Child Tax Credit	1
Visit adherence for child	1	Receipt of Earned Income Tax Credit	1
		Savings	1
		Tax filing status	1
		Tax refund amount	1

^a^ Financial coaching services were delivered in a bundle, including financial coaching, one‐on‐one case management, public benefit screening and referral, community resources screening and referral, assistance in applying or obtaining benefits, budgeting, and goal setting.

**Table 2:** Summary of characteristics by study.

References	Analytic sample size	Study design	Intervention	Outcomes and measurement timing	Results
Marcil & Thakrar (2022a, 2022b)	186	QED	**Setting:** Pediatric primary care clinic **Services:** VITA tax preparation **Sessions:** One in‐person session, unreported length of contact **Comparison condition:** Treatment as usual: Usual care	CTC receipt EITC receipt Tax refund receipt Tax refund amount Measured once following intervention.	Authors reported participants, compared to non‐participants, received larger amounts in Child Tax Credits and Earned Income Tax Credits.
Markowitz et al. (2022)	81	QED	**Setting:** Pediatric medical home **Services:** VITA tax preparation **Sessions:** One in‐person session, unreported length of contact **Comparison condition:** Nothing or waitlist	Tax‐filing Status Measured once following intervention.	Authors concluded that embedding free tax services in a pediatric medical home may change tax‐filing behavior from fee‐for‐service to VITA utilization, thus saving participants money.
Schickedanz et al. (2023)	81	RCT	**Setting:** Pediatric primary care clinic **Services:** Financial Coaching ‐Action plan and goal development ‐Assessment of public benefits eligibility and receipt, savings, credit, debt, and expense reduction opportunities ‐Diaper distribution **Sessions:** 20–40 min sessions delivered at least monthly for six months, with more frequency available if requested by participants. Delivered both in person or remotely via phone call or text. **Length of Treatment:** 4–6 months **Comparison condition:** Treatment as usual: Pediatric care	Income Savings Pediatric Visit Adherence Pediatric Vaccination Schedule Adherence All outcomes reported were measured at 6 months, apart from vaccinations, which were measured at 8 months.	Authors reported that the increase in monthly income and average savings by 6 months was greater for participants than the control group, although not statistically significant. Authors reported that participants had fewer missed visits than the control group (0.46 vs. 107 visits missed; *p* = 0.01) for the period from the 1‐month to 6‐month visit recommended by the AAP and fewer missed vaccinations (2.52 vs. 3.98; *p* = 0.002).
Sadigh et al. (2022)	40	RCT	**Setting:** Adult outpatient specialty clinic **Services:** Out‐of‐pocket cost communication Motivational financial navigation Financial counseling **Sessions:** 1 h sessions delivered once at baseline, and once at a 3‐month follow‐up. Delivered remotely via phone call or Zoom. **Length of Treatment:** 3 months **Comparison condition:** Treatment as usual: routine neurology visits, use of available ancillary staff, and internal or external resources for co‐pay assistance or free medication per normal clinic procedures.	Financial worry Material Hardship Physical Quality of Life Mental Quality of Life Care Non‐Adherence All outcomes were measured at 3 months.	*g* = 0.56, CI: −0.06, 1.18 OR = 1 CI: 0.29, 3.48 *g* = −0.27; CI: −0.99, 0.35 *g* = 0.02; CI: −0.59, 0.63 OR = 0.8; CI: 0.23, 2.79

#### 4.1.3 Study characteristics

Marcil and Thakrar (2022a, 2022b) used a QED to determine the benefits of using a VITA tax‐preparation clinic operated through a non‐profit organization known as StreetCred. The VITA site was embedded in a pediatric primary care setting, which was located at the Boston Medical Center (BMC). StreetCred began as a pilot study in the Boston Medical Center in 2016, and expanded nationally between 2018 and 2019 (Boston Medical Center, n.d.). The primary goal of the StreetCred program is to offer financial coaching and tax preparation services (Boston Medical Center, n.d.). Outcome measures included the receipt of the CTC, receipt of the EITC, whether participants received a tax refund, and if so, the amount of that refund. StreedCred was offered to parents of BMC pediatric patients, adult BMC patients, and community members from January through July of 2021. Of the 325 individuals who used StreetCred at the site for the tax season, 186 were study participants. Eighty‐eight parents of pediatric patients of the BMC were in the treatment group and thereby consented to their tax data being used for the study, and completed the research survey. The control group included parents of BMC pediatric patients who verbally consented to and completed a control tax survey between the months of July and October 2021 after the tax preparation season had passed (*n* = 98).

Markowitz et al. (2022) used a QED to determine the benefits of a VITA tax‐preparation clinic, operated through StreetCred New Haven, embedded in a pediatric medical home. StreetCred New Haven, an expansion of The Boston Medical Center program, provided tax preparation services only for the purposes of this study. Participants of the study were required to be the primary caregiver of a pediatric medical home patient. The intervention outcomes reported included tax‐filing status. Study authors also explored participants' knowledge about StreetCred/VITA services and attitudes about the pediatric medical home as a location for VITA services and help with financial issues.

Participants for this study were recruited from the waiting room as they were waiting to have their taxes filed by the StreetCred New Haven program. A research assistant was present for 31 of the 33 tax preparation sessions that were offered during the time period of the intervention, which took place from February 7th to April 10th, 2019. All tax filers in the waiting room on the days the research assistant was present were asked if they were interested in participating in the study. Both intervention group and control group participants completed a research survey after they consented to participate. The intervention group was made up of participants who consented to participate in the study, and who were the primary caregivers of a patient of the pediatric medical home (*n* = 20). The control group participants were recruited from May 1st to July 9th, 2019, after the tax filing deadline through StreetCred was closed. Control group participants were also caregivers to patients of the PMH and did not use StreetCred to file their taxes (*n* = 61). No attrition or missing data was reported for either group. After completing the survey, control group participants were provided with $10 and information sheets including financial capability services available in the community.

Schickedanz et al. (2023) used a randomized control trial research design to determine the benefits of financial coaching on outcomes related to financial and health‐related goals. Primary outcomes reported by the study included income, savings, debt, pediatric visit adherence, and vaccination visit adherence. The participants were recruited and enrolled from the waiting area or the examination room of a pediatric primary care clinic, between fall and summer. Once consent for the study was obtained, research staff randomized participants to either the intervention or control group using a computer‐generated simple randomization. Participants were compensated $20 incentive cards at enrollment, and again at the 6 month follow‐up. Eighty‐one participants were randomized into intervention or control groups, with no attrition throughout the study. Of the 81 participants, 35 were allocated to the intervention group, and 46 were allocated to the control group.

The control group in the Schickedanz et al. (2023) study received care as usual per clinic practices. This included visit reminders through phone calls, text messages, and mailed postcards. Control group participants also received a paper screening for social needs as part of typical clinic protocol, which was reviewed by their healthcare provider, and referrals to external resources were then provided.

Sadigh et al. (2022) used a randomized control trial research design to explore the effects of integrating a remote financial navigation and counseling program known as CostCOM into an adult specialty care clinic site serving Multiple Sclerosis patients. Outcomes measured included financial worry, material hardship, physical quality of life (QOL), mental QOL, and care non‐adherence. The study identified 73 patients who were eligible for participation. Of the 73, 11 declined to participate in the program. Participants who consented to participate in the study were randomized by initially being assigned a case number. Blocked randomization was used via a randomization table created by a biostatician involved in the study. After consent was obtained for participation, the study coordinator contacted the principal investigator of the study to determine the group assignment for each participant.

The total number of participants randomized to either the intervention or control group was 62, with 32 being allocated to the intervention group and 30 being allocated to the control group. Of the 32 that were allocated to the intervention group, 1 dropped out of the study before receiving the intervention, and 8 discontinued the intervention. This resulted in an analytic sample size for the intervention group of 22. For the 30 participants allocated to the control group, 3 participants dropped out before the start of the intervention, and 9 discontinued after the start of the intervention period. This resulted in a final analytic sample size of 18 participants for the control group. The total analytic sample size for the study was 40 participants.

Control group participants in the Sadigh et al. (2022) study received usual care, which consisted of routine neurology visits, use of available ancillary staff, and use of existing internal or external resources for co‐pay assistance or free medication.

#### 4.1.4 Participant characteristics

The target participants were primarily adults, with three of the four studies focusing on adult participants (Marcil & Thakrar, 2022a, 2022b; Markowitz et al., 2022; Sadigh et al., 2022) and one study focusing on adult and child dyads (Schickedanz et al., 2023).

Across the four studies, participants were primarily female, with female participants comprising 80% or greater of each sample. Participants in the Schickedanz et al. (2023) study were all low‐income, Marcil and Thakrar (2022a, 2022b) and Markowitz et al. (2022) did not report income level, and Sadigh et al. (2022) reported annual income for participants as either above or below $60,000. The three studies that reported mean ages had adult‐only participants, with the average age range of participants in these studies being 30 to 41.5 years old. The predominant race for three of the studies was African American (Marcil and Thakrar, 2022a, 2022b; Markowitz et al., 2022; Sadigh et al., 2022). The Schickedanz et al. (2023) study did not specify the number of African American participants, and only reported the percentage of White participants (50%), and those identifying as Hispanic (50%). Following African Americans, White participants made up most of the sample for each study, ranging from 10% to 50% of participants. One study (Schickedanz et al., 2023) reported a sample in which half of the participants were Hispanic, with the remaining three studies reporting Hispanic participants as being between 11% and 30% (Marcil & Thakrar, 2022a, 2022b; Markowitz et al., 2022; Sadigh et al., 2022).

#### 4.1.5 Intervention characteristics

##### Services provided

Two of the studies (Marcil and Thakrar, 2022a, 2022b; Markowitz et al., 2022) focused on tax preparation only as their financial intervention, and both studies offered a VITA tax clinic on‐site in the healthcare clinic setting. In another study (Schickedanz et al., 2023), services were labeled as financial coaching, which was further specified as one‐on‐one case management, public benefits screening and referral, community resource screening and referral, assistance in applying or obtaining benefits, budgeting, and goal setting. Though Sadigh et al. (2022) did not label their services as financial coaching, Schickedanz et al. (2023) and Sadigh et al. (2022) overlapped in their offering of public benefits screening and referral, community resources and referral, and assistance in applying or obtaining benefits and resources. Though there was overlap in services provided, Schickedanz et al. (2023) had more frequent contact and placed an emphasis on fostering a longitudinal client‐coach relationship, while frequency of contact between coaches and participants in the Sadigh et al. (2022) study was limited to two sessions. Schickedanz et al. (2023) also delivered services in person during clinic appointments, while Sadigh et al. (2022) delivered sessions remotely.

Markowitz et al. (2022) utilized an MFP called “StreetCred New Haven,” which offers VITA services for free tax preparation as its primary financial service. The program was advertised from September through April 2019 via flyers and written in after‐visit summaries, and advertised verbally through referrals from the healthcare team, financial capability organizations, and a 2‐1‐1 information line. From January to April 2019, 33 tax preparation sessions were held during and after clinic hours. The intervention was completed in one session, and the average time needed to complete the session was not reported.

Marcil and Thakrar (2022a, 2022b) utilized StreetCred to offer a VITA site for free tax preparation services at the Boston Medical Center pediatric clinic. Additional information about recruitment, referral procedures, and services were not available for this study. The intervention was completed in one session, and the average time needed to complete the session was not reported.

Training received by those delivering the intervention for both studies (Marcil & Thakrar, 2022a, 2022b; Markowitz et al., 2022) was consistent for the identified intervention of tax preparation. All VITA tax sites are government or public‐funded, and are composed of non‐profit volunteers who are certified by the IRS to deliver tax preparation assistance.

Schickedanz et al. (2023) provided financial coaching to the primary caregivers of infants during their well‐child visits. This intervention typically took place while caregivers were waiting for their child's appointment to begin, and the average length of visit was between 20 and 40 min. Each participant was paired with a coach at enrollment. Participants were then followed up with at least monthly, either at their child's pediatrician visit or remotely via phone calls or text messages. Additional contact was provided to participants who expressed a need or interest. The financial coaching intervention used was modeled after a program known as LIFT, which is a national financial coaching organization. Researchers reported that this organization was the study's community partner organization. This intervention approach was reported as utilizing a longitudinal relationship between the client and their coach to build trust and motivation, with the goal of fostering client‐directed financial behavioral change to meet their financial goals, strengths, and needs.

The bundle of services provided by Schickedanz et al. (2023), was labeled as financial coaching. This intervention included several services, beginning with the development of goals and action plans through motivational interviewing. The service also included an assessment of income, public benefits eligibility and receipt, savings, credit, debt, taxes, and potential for expense reduction opportunities. Coaches also helped participants navigate through available services such as affordable childcare, transportation and utility discounts, nutrition assistance, and free tax preparation. An additional service provided was written referral materials and diapers when they were requested by participants.

The intervention in the Schickedanz et al. (2023) study was provided by social work students who were supervised by a licensed clinical social worker. The social work students delivering the intervention received 16 h of additional training in financial coaching delivered by LIFT before working with participants, and received continuing education in motivational interviewing, social determinants of health, community partnerships, and integrated healthcare throughout the course of the intervention.

Sadigh et al. (2022) collaborated with a private organization, TailorMed Medical Inc., to develop an intervention referred to as CostCOM, which included out‐of‐pocket cost communication, motivational financial navigation, and financial counseling. This intervention was delivered remotely via phone calls or Zoom. Out‐of‐pocket cost communication consisted of a review of the patient's insurance and predicted costs for their treatment, with a total estimate being offered at baseline and again at 3 months if the participant had any insurance or treatment changes. Financial navigation was defined as professional guidance to identify financial service programs, with examples provided being copay and living expenses that alleviate costs of care. Participants were also offered information about how to improve their insurance coverage. The financial counseling piece of the intervention was to address participants' concerns about finances and to enroll them in financial assistance programs for which they were eligible. Services were provided by a remote financial counselor at TailorMed in two, 1 h sessions, one which took place at enrollment, and one taking place at a 3 month follow‐up. The total length of treatment was 3 months, with a total of two contacts.

### 4.2 Risk of bias in included studies

The risk of bias varied across studies from “some concerns” to “critical.” None of the four studies had a low risk of bias. Three of the four included studies had pre‐registered protocols, which was helpful in assessing several domains, including deviations from intended interventions, measurement of outcomes, and in selection of the reported result reporting bias. The studies that used an RCT design differed in their risk of bias on almost all of the domains, with both having a low risk of bias for measurement of outcomes. Alternatively, the studies that used a QED design had virtually identical assessments of risk of bias across all of the domains, with the exception of selection of reported results. Study authors recognized study limitations and recommended that research on intervention effectiveness continues, and that rigorous research be conducted on the MFP interventions.

#### 4.2.1 Risk of bias details

As seen in Table 4, two studies were randomized control trials (RCT) and were evaluated using the RoB 2 (add cite). The first, a study by Schickedanz et al. (2023), was an RCT with low risk of bias for all domains, with the exception of the randomization process, which was judged “some concerns.” This study lacked an explicit statement that staff were unaware of the allocation process. Therefore, the overall risk of bias was judged “some concern.” The second RCT study, Sadigh et al. (2022), was judged low risk for all but three domains. The study was deemed “high risk” of bias for two domains; deviations from intended interventions (due to respondents, carers and staff delivering the intervention were aware of assignment to condition, and study dropouts may have inflated the intervention effects), and missing outcome data (outcome data missing on study dropouts). The study was also judged as having “some concern” for the selection of reported results (there are significant reporting changes from the protocol to the reported results). The overall study was judged as having a high risk of bias.

The two remaining studies used QEDs, and were therefore assessed using the ROBIN‐I tool. Markowitz et al. (2022) was found to have a non‐low risk of bias for three domains: (1) confounding (lack controls for group race/ethnicity differences) (serious risk of bias); (2) selection of participants (groups selected at different times and recruited differently) (critical risk of bias); and (3) missing data (treatment outcome data not reported) (serious risk of bias). The overall study risk of bias was deemed “critical.” The study by Marcil and Thakrar (2022a, 2022b), was found to have low risk of bias with the exception of four domains: (1) confounding (due to lack of controls for ethnicity differences across groups) (serious risk of bias); (2) selection of participants (the selection of participants was related to the outcome) (critical risk of bias); (3) missing data (unclear outcome data) (serious risk of bias); and (4) selection of the reported result (multiple analyses of intervention outcome relationship due to absence of reporting health outcomes) (serious risk of bias). The overall judgment was that the study has a “critical” risk of bias.

### 4.3 Data and analysis

#### 4.3.1 Financial outcomes

All four included studies measured at least one financial outcome; however, they varied in the types of financial outcomes measured. Two studies measured tax filing status, one measured CTC and EITC amounts, one measured household income and savings, and one measured material hardship and financial worry. For Marcil and Thakrar (2022a, 2022b) and Markowitz et al. (2022), both interventions focused on tax preparation and filing, measured tax filing status and Marcil and Thakrar (2022a, 2022b) also measured CTC and EITC amounts. Markowitz et al. (2022) did not report sufficient outcome data to calculate effect sizes, but Marcil and Thakrar (2022a, 2022b) did provide sufficient data to calculate tax filing status, but not CTC or EITC amounts. Markowitz et al. (2022) concluded that embedding free tax services in a pediatric medical home may change tax‐filing behavior from fee‐for‐service to VITA utilization, thus saving participants money. In the Marcil and Thakrar (2022a, 2022b) study, the effect on tax filing status was small and not statistically significant (OR = 1.08 L CI: 0.51, 2.26) and authors reported participants, compared to non‐participants, received larger amounts in CTCs and EITCs. Schickedanz et al. (2023) reported household income and household savings measured via participant self‐report. They reported the participants' increase in monthly income and average savings by 6 months was greater than control participants, although not statistically significant. Sadigh et al. (2022) reported two financial‐related outcomes at 3 months, material hardship measured via self‐report, and financial worry measured using the Comprehensive Score for Financial Toxicity (COST) measure. The author reported sufficient data to calculate effect sizes. The effects on financial worry (Hedges' *g* = 0.56, CI: −0.06, 1.18) and material hardship (OR = 1 CI: 0.29, 3.48) were not statistically significant.

#### 4.3.2 Health‐related outcomes

Two of the studies reported health‐related outcomes (Sadigh et al., 2022; Schickedanz et al., 2023). Sadigh et al. (2022) measured care non‐adherence via self‐reported positive responses to delaying, foregoing, stopping, or changing prescribed medication due to cost, and physical and mental health QOL using the Patient‐Reported Outcomes Measurement Information System (PROMIS)−10. Effects for care non‐adherence (OR = 0.8; CI: 0.23, 2.79), physical health QOL (Hedges' *g* = −0.27; CI: −0.99, 0.35), and mental health QOL (Hedges' *g* = 0.02; CI: −0.59, 0.63) were small and not statistically significant. Schickedanz et al. (2023) examined missed preventive care visits and missed or delayed vaccinations. Authors reported that participants had fewer missed visits than the control group (0.46 vs. 107 visits missed; *p* = 0.01) for the period from the 1‐month to 6‐month visit recommended by the AAP and fewer missed vaccinations (2.52 vs. 3.98; *p* = 0.002).

#### 4.3.3 Excluded studies

Of the 25 studies that were screened at the full text screening stage, we excluded 20 studies from the review. Most were ruled out due to incorrect study design. Some studies were excluded for multiple reasons. We have included the primary reason for exclusion in the table. Two studies were excluded because we could not access them. See Table 3 for a list of excluded studies.

**Table 3:** Reasons for exclusion.

Study number	References	Reason for exclusion
1	Beck et al. (2018)	Not RCT or QED
2	Bell et al. (2020)	Not RCT or QED
3	Berkowitz et al. (2017)	Not RCT or QED
4	Black et al. (2020)	Not RCT or QED
5	Bovell‐ammon et al. (2023)	Wrong intervention
6	Dayan et al. (2023)	Outside US
7	Doust (2014)	Cannot access
8	Fierman et al. (2016)	Not RCT or QED
9	Knight et al. (2022)	Not RCT or QED
10	Langford (2013)	Not RCT or QED
11	Linney (1990)	Not RCT or QED
12	Marcil et al. (2018)	Not RCT or QED
13	Nadler and Schimmel (1991)	Not RCT or QED
14	Oostra (2022)	Not RCT or QED
15	Politi et al. (2021)	Wrong intervention
16	Santos et al. (2023)	Not RCT or QED
17	Shankaran et al. (2018)	Not RCT or QED
18	Sherman (2017)	Not RCT or QED
19	Watabayashi et al. (2020)	Not RCT or QED
20	Wegner (1993)	Cannot access

Abbreviations: QED, quasi‐experimental design; RCT, randomized controlled trial.

**Table 4:** Summary risk of bias.

Type of bias	Judgment	Support for judgment
**Study Name:** Schickedanz et al. (2023) (RoB 2)		
Randomization Process	Some concerns	No explicit statement that staff were unaware of method of assignment to group assignment
Deviations from intended interventions	Low	No deviations from intended intervention
Missing outcome data	Low	No missing outcome data
Measurement of outcome	Low	Measurement consistent with protocol
Selection of reported result	Low	No selection of reported results
**Overall**	**Some concerns**	
**Study Name:** Sadigh et al. (2022) **(RoB 2)**		
Randomization Process	Low	Random assignment to groups
Deviations from intended interventions	High	Respondents, carers and staff delivering intervention aware of assignment to condition, and study dropouts may have inflated effect
Missing outcome data	High	All outcome data missing on study dropouts
Measurement of outcome	Low	Measurement consistent with protocol
Selection of reported result	Some concerns for all outcomes	Significant reporting changes from protocol to study
**Overall**	**High**	
**Study Name:** Markowitz et al. (2022) (ROBINS‐I)		
Confounding	Serious	Controls for group race/ethnicity differences absent
Selection of participants	Critical	Confounding variables (treatment and comparison groups selected at different times) and selection bias concerns (groups recruited differently)
Classification of interventions	Low	Intervention well defined
Deviations from intended interventions	Low	No deviations from intended interventions
Missing data	Serious	Treatment outcome data not reported
Measurement of outcomes	Low	No deviations of outcomes measurement reported
Selection of reported result	Low	No selection of reported results
**Overall**	**Critical**	
**Study name:** Marcil & Thakrar (2022a, 2022b) (ROBINS‐I)		
Confounding	Serious	Lack controls for ethnicity group differences
Selection of participants	Critical	Selection of participants related to outcome
Classification of interventions	Low	Intervention well defined
Deviations from intended interventions	Low	No deviations from intended intervention
Missing data	Serious	Unclear outcome data
Measurement of outcomes	Low	No deviations of outcomes measurement from protocol
Selection of reported result	Serious	Multiple analyses of intervention outcome relationship
**Overall**	**Critical**	

## 5 DISCUSSION

### 5.1 Summary of main results

The challenges of low income and poverty impacts both the financial well‐being and the health of Americans (US Census Bureau, 2022; US Department of Health and Human Services, n.d.). Increasingly, healthcare systems are seeking to address financial stress as a social determinant of health by incorporating financial services within the healthcare delivery model (Francis et al., 2018; World Health Organization, 2023), called an MFP. The purpose of this systematic review was to determine the state of research and examine the health and financial outcomes of MFPs.

This review included a total of four unique studies reported on in five reports. The studies reported on a range of services provided within an MFP, and in several types of healthcare settings. Two of the four included studies provided free tax preparation services, public benefits screening and referral, and/or community resources benefits and referrals. The remaining two studies provided other services. One study provided financial counseling, and one provided financial coaching, including one‐on‐one case management, budgeting, and goal setting.

Overall, the evidence of the health and financial effects of MFPs is sparse, given the small number of studies using randomized or QEDs. Moreover, the lack of standardization of outcomes studied, measurement of the outcomes, or timing of measurement did not allow for synthesis of outcomes across studies. None of the studies examined effects of similar types of interventions on similar outcomes, and several were missing sufficient data to calculate effect sizes, thus we were not able to pool effects of studies to conduct a meta‐analysis.

Results indicate small and nonsignificant effects of MFPs on financial outcomes reported, and some author‐reported positive statistically significant effects on attending appointments and vaccination schedule adherence.

### 5.2 Overall completeness and applicability of evidence

We were able to calculate the effect sizes for all reported outcomes in only one study (Sadigh et al., 2022) and one outcome in a second study (Marcil & Thakrar, 2022a, 2022b). We requested missing outcome data from authors, but authors did not provide the missing outcome data. Due to the absence of data, we were unable to pool the data for meta‐analysis. Collectively, the four studies lacked evidence for longer term financial effects (longer than 6 months), a variety of doses (beyond two visits), and varying length of treatment (length for 50% of the studies was unspecified). All studies suffered from methodological flaws that limit the validity of results. Thus, the evidence provided about the MFP intervention from the included studies lacks completeness to be generalizable related to the type of settings, intervention components, geography, and household income.

In terms of settings, three of the four studies resided in pediatric primary care settings, and one resided in specialty adult care clinic (neurology). Thus, non‐pediatric primary care settings, other types of adult speciality care settings, and tertiary settings were not represented in these studies. Relative to MFP components, half of the included studies provided only on‐site VITA free tax assistance (Marcil & Thakrar, 2022a, 2022b; Markowitz et al., 2022), one offered financial coaching with bundled services (Schickedanz et al., 2023), and the fourth offered a package of financial services that did not include financial coaching (Sadigh et al., 2022). Given the lack of standardization, generalization about MFP intervention components is difficult. Other potential financial services, or combinations of services, were not included among the studies. The lack of geographic representation across the United States also presents a challenge to generalization. Two studies were located on the east coast (Marcil & Thakrar, 2022a, 2022b in New Haven, CT, and Markowitz et al., 2022 in Boston, MA), one was located in Los Angeles, CA (Schickedanz et al., 2023), while the location of the fourth (Sadigh et al., 2022) was unknown. The lack of known representation outside of the U.S. east and west coast also limits generalizability.

Other aspects of the studies also provide challenges for generalization. The sample sizes among the studies were mostly small, with three of the four studies presenting sample sizes smaller than 100 total. Larger sample sizes are desirable to achieve sufficient power in the analysis to detect smaller effects that may be missed in smaller sample studies and for accuracy. The studies may have mostly targeted a low‐income population, but information is limited. One study (Schickedanz et al., 2023) specified that subjects were from low‐income backgrounds, but the Sadigh et al. (2022) article reported findings relative to $60,000 annual income, while the other two (Marcil & Thakrar, 2022a, 2022b) and Markowitz et al., 2022) did not specify (although the intervention featured in these studies – VITA free tax preparation – targets a lower‐income population). The subjects of three of the four studies were predominantly African American, and therefore generalizability to other populations would be challenging.

### 5.3 Quality of the evidence

While each of the four included studies provide evidence of the effectiveness of the intervention, the evidence across the studies is incomplete due to outcome reporting issues, methodological weaknesses and important concerns regarding risk of bias. While two of the four included studies used random assignment, and three of the four studies had registered protocols, there was little consistency in the outcomes measured, given that each collected data on a different set of health and financial outcomes.

While two of the studies used random assignment, all of the studies contained important methodological weaknesses. For example, two of the four studies assessed for fidelity, but none of the studies reported the outcome of the fidelity assessment. Although three of the four studies had registered protocols, all of the studies were assessed as “non‐low” risk of bias due to various factors. In addition, two studies did not assess for pretest differences on outcomes. Given the lack of data to calculate effect sizes, inability to perform a meta‐analysis, risk of bias issues, and methodological weaknesses, we conclude that evidence is sparse about the effectiveness of the MFP intervention on health and financial outcomes.

The studies included here have important strengths. Research design is a strength for several studies. For instance, two of the four studies are RCT's (*k* = 2), and Sadigh et al. (2022) study examines five financial and health outcomes across 3 months. In addition, all of the studies used a manualized intervention. Another strength is related to training – three of the four studies provided training for the staff providing the intervention, either from the IRS (Marcil & Thakrar, 2022a, 2022b; Markowitz et al., 2022) or an organization (LIFT) (Schickedanz et al., 2023). Both RCTs and one QED had registered protocols.

The risk of bias varies across studies. Two different tools were used to assess the risk of bias – the RoB 2 tool for the two RCT studies, and the ROBIN‐I for the two QED studies. One RCT study was assessed as “some concerns” due to the randomization process, and the second was assessed as“high risk” due to deviations from the intended intervention, missing outcome data and selection of reported result. Two QED studies were assessed as “critical” risk of bias due to confounding, selection of participants, and missing data, and in addition, one of the studies had concerns about selection of reported results. While the number and quality of studies included in this review precludes providing a clear answer on the health and financial effects of MFPs, this review provides a good indicator of the state of the evidence for this intervention and identifies important gaps to inform future research.

### 5.4 Potential biases in the review process

The conduct and reporting of this review were guided by Campbell's standards and policies for the conduct and reporting of systematic reviews to ensure a rigorous and transparent review designed to minimize bias and error (Campbell Collaboration, n.d.). To limit bias in the review process, we conducted a thorough and transparent search for published and unpublished studies and conducted a rigorous process for screening and selecting studies and extracting data using at least two independent reviewers. We also included and reported all financial and health outcomes relevant to this review per the protocol. This review is not without its limitations, however, and the findings must be interpreted in light of the study's limitations. While we made every attempt to search for published and unpublished studies, all of the studies that met inclusion for this review were published journal articles. Because we limited studies to those conducted in the United States and were published in English due to the uniqueness of the U.S. healthcare and financial systems, our findings are limited to the United States context.

Despite these limitations, this review provides a comprehensive summary of MFPs that combine financial services and healthcare services. This is the first systematic review examining health and financial outcomes of MFPs, and therefore sheds early light on the promise and limitations of the body of evidence of MFPs on financial and health outcomes, as well as gaps to inform future MFP interventions development and research.

### 5.5 Agreements and disagreements with other studies or reviews

This is the first systematic review of MFP interventions on health and financial outcomes.

Therefore, we are unable to comment on the degree to which this review agrees with similar reviews or studies.

## 6 AUTHORS' CONCLUSIONS

### 6.1 Implications for practice and policy

Due to the small number of included studies, the lack of sufficient data to calculate the effects on health and financial outcomes, the diversity of components and services offered and the non‐low risk of bias across the studies indicates a lack of strong evidence about the effectiveness of MFP interventions. Although MFP interventions are increasingly offered, the lack of strong evidence suggests that, while many see these as promising interventions, more evidence is needed to fully support more wide‐scale implementation.

The lack of strong evidence about the effectiveness of MFP interventions has implications for practice and policy in several ways. First, the diversity of components of MFP interventions results in challenges to comparing processes and outcomes across studies. The diversity of components also results in interventions being delivered by practitioners from various professional backgrounds and in different types of healthcare settings with various target audiences and goals. However, practitioners seek evidence on the effectiveness of their choice of components with MFPs so they can deliver the most effective and efficient intervention towards their healthcare and financial goals. The lack of evidence across MFPs points to the need to develop a more definitive evidence base about the components that provide the desired outcomes.

Policy actors in the healthcare system that seek to facilitate improved health outcomes through the MFP intervention can seek a stronger evidence foundation for the effectiveness of MFP interventions. Policy related to the MFP intervention in each type of setting (primary, specialty care, and tertiary), component set, and outcomes measured, resides within healthcare organizations that govern healthcare sites. These organizations, collectively, can promote standardization of component sets within specific types of settings, as well as outcome measures. Standardization of measurement time points post‐intervention, as well as other aspects of the intervention, would assist in the development of an evidence base. Organizational policy work can facilitate additional research on MFPs using strong methodology, manualized MFP interventions, and common outcomes to assist in building a strong evidence base.

### 6.2 Gaps in practice settings

All four studies included in this review took place in outpatient clinic settings. Little research has explored whether individuals may benefit from MFPs located in other settings, such as inpatient and home settings. Due to the nature of the privatized healthcare system in the United States, financial stress may be a concern for those experiencing inpatient hospitalization. There may be an opportunity to explore the benefit of an MFP designed to assist individuals who are hospitalized, especially for those with chronic illness and their family members. Individuals who experience chronic illness may experience frequent hospitalizations that can lead to reduced income due to the physical symptoms of having a chronic illness, lost wages from employment due to absence, and medical bills from treatment (Ghazal et al., 2023). Current care as usual for these patients includes outside resource referral to community resources and to hospital financial services, which may alleviate the financial stress related to medical care and costs. However, hospital staff often do not have specific skills tailored to addressing the full scope of financial needs of their patients. MFP providers would have the opportunity to build long‐term relationships with these patients and their caregivers, given the nature of repeated or long‐term inpatient hospitalization, and therefore MFP interventions may provide the financial benefits desired.

Likewise, little research has been conducted on the use of MFPs combined with home health and visiting nurse settings. Home visits by nurses and other trained medical personnel have had positive health and social outcomes for medically and socially vulnerable populations, including low‐income families and seniors (Lizano‐Díez et al., 2022; Minkovitz et al., 2016). Typically, these services provide minimal material assistance such as infant or adult diapers, infant care supplies, and/or basic home health monitoring equipment. All other services, including financial assistance or coaching, are provided as referrals. MFPs made in conjunction with an established home health or visiting nurse model may achieve the desired financial benefits from the established ease of client access (due to previously established routine of home visits) and increased levels of trust and engagement afforded by associating with a client's known healthcare team.

### 6.3 Implications for research

The systematic review highlights several gaps in the evidence base for MFPs, pointing to important implications for future research. The limited number of studies, combined with the diversity of MFP components and services and the lack of consistent outcomes, underscores the need for a more rigorous and comprehensive approach to MFP intervention research. Future research should prioritize the clear description of MFP components and consider assessing different components separately within research designs to identify what works most effectively. This approach will enable a more nuanced understanding of how specific components contribute to health and financial outcomes. One of the key challenges in MFP research we identified is the lack of consistency in the outcomes measured across different studies. Developing a common core of outcomes for MFP interventions is essential to enable meaningful comparisons and meta‐analyses. This core set of outcomes should include both health‐related and financial measures that are universally relevant across diverse healthcare settings. By standardizing outcome measures, researchers can generate more robust evidence on the effectiveness of MFPs and identify the components that yield the most significant benefits. Another notable issue that could be addressed in future research is the lack of diversity of settings in which MFP interventions were implemented. This review found that all included studies were conducted in outpatient clinic settings, leaving a significant gap in understanding the potential benefits of MFPs in other healthcare environments. Future research should explore the implementation of MFPs in inpatient and home‐based care settings, where financial stress may be more acute due to chronic illness and prolonged hospitalization. Investigating the impact of MFPs in these settings could provide valuable insights into how such interventions can be adapted to meet the needs of diverse patient populations and healthcare contexts. Finally, more and better research is needed in this area. Enhancing methodological rigor, including the use of RCTs and standardized time points for outcome measurement, will be crucial to building a strong evidence base.

## CONTRIBUTIONS OF AUTHORS

All authors contributed to the conduct of the review and preparation of the manuscript.

Content: Julie Birkenmaier, Harly Blumhagen, Hannah Shanks.

Systematic review methods: Brandy Maynard, Julie Birkenmaier, Hannah Shanks, Harly Blumhagen.

Statistical analysis: Brandy Maynard, Harly Blumhagen.

Information retrieval: Brandy Maynard, Harly Blumhagen, Hannah Shanks, Julie Birkenmaier, with expert guidance from Rebecca Hyde.

## DECLARATIONS OF INTEREST

No authors have any conflicts of interest.

## PLANS FOR UPDATING THIS REVIEW

The review authors do not have current plans for updating this review.

## DIFFERENCES BETWEEN PROTOCOL AND REVIEW

In a deviation from the protocol, we did not search Conference Proceedings and Citations Index since we did not have institutional access; however, Google Scholar includes conference proceedings. In another deviation from the protocol, we searched the website Social Interventions Research and Evaluation Network (SIREN) by the University of San Francisco California.

Following the estimation of individual study‐level effects, we had planned to conduct a separate meta‐analyses to pool studies for each outcome construct. Given the lack of effect size data and lack of standardization of outcomes measured, we were unable to do so.

## SOURCES OF SUPPORT

### 1 Internal sources

This study had no internal sources of support.

### 2 External sources

This study had no external sources of support.

## CHARACTERISTICS OF STUDIES AWAITING CLASSIFICATION

None.

## CHARACTERISTICS OF ONGOING STUDIES

The Schickedanz et al. (2023) study (NCT037365590) is ongoing. In a communication to the review authors, study authors state that they plan to issue a publication on the full set of impacts (only partial impacts reported in the included study here), as well as a 2‐year outcome report.

## DATA AND ANALYTIC CODE

All extracted data, including effect sizes, is available as supplementary material from Wiley Online Library.

## Supporting information

Supporting information.

Supporting information.

Supporting information.

Supporting information.
