# Correction to “Medical‐Financial Partnerships for Improving Financial and Medical Outcomes for Lower‐Income Americans: A Systematic Review”

**DOI:** 10.1002/cl2.70066

**Published:** 2025-09-16

Birkenmaier, J., B. R. Maynard, H. M. Blumhagen, and H. Shanks. 2024. “Medical‐Financial Partnerships for Improving Financial and Medical Outcomes for Lower‐Income Americans: A Systematic Review.” *Campbell Systematic Reviews* 20: e70008.

https://doi.org/10.1002/cl2.70008.

Below reference has been added to the reference list as well as cited in text in Section 4.2.1. It was missing in the originally published article.

Sterne, J. A. C., J. Savović, M. J. Page, et al. 2019. “RoB 2: A Revised Tool for Assessing Risk of Bias in Randomised Trials.” *BMJ* 366: l4898.

We apologize for this error.

