# Supplementary material for: Medical‐financial partnerships for improving financial and medical outcomes for lower‐income Americans: A systematic review
Source: Campbell Syst Rev. 2024 Dec 6;20(4):e70008. doi: 10.1002/cl2.70008 (PMC11621975; doi:10.1002/cl2.70008)
Supplement: Supplementary file 1 — Supporting information. [file CL2-20-e70008-s003.docx]

**Peer Review Feedback**

**Campbell Collaboration**

**SWCG-23-04**

**Title of review:** Medical-financial partnerships for improving financial and health outcome for lower-income Americans:  A systematic review

|  | **Peer Review comments** | **Editorial Comments** | **Author’s response** |
| --- | --- | --- | --- |
| **GENERAL POINTS** Campbell accepts non-intervention reviews such as reviews of outcome measures, risk/protective factors, methods, qualitative evidence and overviews or review of reviews.  The review should be written in language that is easily understood. | **Peer Reviewer 1**: Nothing to declare  **Peer Reviewer 2**: This paper was thorough and detailed in its methods and approach. While the number of studies that met the inclusion criteria were extremely low (4), it remains a worthwhile contribution to the literature and offers future health services researchers and clinical implementers useful insight into the sort of research that remains needed.  **Methods**: I don't have any additional concerns. |  |  |
| **TITLE**  (Only comment if you strongly disagree with the current title) | **Peer Reviewer 1**: Nothing to declare  **Peer Reviewer 2**:  **Methods**: |  |  |
| **ABSTRACT**  All headings are included (i.e., background, objectives, search methods, selection criteria, main results, conclusion) | **Peer Reviewer 1**: In the “search methods” item, authors state: “Following the procedures outlined in the protocol”. This is the abstract; thus, readers don’t have access to the protocol. Without knowing the protocol, it is difficult to understand the sentence. Suggestion: Using selected terms, studies were retrieved from Google….  **Peer Reviewer 2**:  **Methods**: |  | We have edited the abstract to “Using the selected terms,...” |
| **PLAIN LANGUAGE SUMMARY**  The summary should explain the topic and results clearly | **Peer Reviewer 1**: Nothing to declare  **Peer Reviewer 2**:  **Methods**: |  |  |
| **BACKGROUND** (Approved at protocol stage; only comment if you have serious concerns) | **Peer Reviewer 1**: Nothing to declare  **Peer Reviewer 2**:  **Methods**: |  |  |
| **OBJECTIVES**  (Approved at protocol stage; only comment if you have serious concerns) | **Peer Reviewer 1**: Nothing to declare  **Peer Reviewer 2**:  **Methods**: |  |  |
| **CRITERIA FOR CONSIDERING STUDIES** (Approved at protocol stage; only comment if you have serious concerns) | **Peer Reviewer 1**: Although part of the approved protocol, the criteria of “on-site interventions” is limiting. Patients may be referred to community resources, close to their residences or close to their work, which may be considered more convenient or trusted/reliable than in a healthcare setting. Additionally, not all healthcare settings have the space to accommodate such interventions and partnering with places that offer MFPs might be a feasible solution, especially if recommended/referred by a trusted heath care personnel/staff/provider.  **Peer Reviewer 2**:  **Methods**: |  | In “The Intervention” section on pg 7, we explain that the model differs from a more traditional medical model of referral to an off-side service. We state, “Medical Financial Partnerships (MFPs), a relatively new and innovative intervention, are currently being piloted within healthcare settings that serve lower-income and Medicaid-insured patients.” Therefore, our inclusion criteria was consistent with the model under review. The reviewer is referring, instead, to a referral model, rather than an MFP model. |
| **SEARCH METHODS AND SOURCES**  Search strategy is comprehensive and appropriate for the topic | **Peer Reviewer 1**: Nothing to declare  **Peer Reviewer 2**:  **Methods**: |  |  |
| **METHODS** (Approved at protocol stage; only comment if you have serious concerns, or if authors have commented on changes to the original protocol, e.g., have the authors adequately justified such changes.) | **Peer Reviewer 1**: As mentioned above, inclusion criteria is limiting.  **Peer Reviewer 2**:  **Methods**: |  |  |
| **RESULTS** This section and the following sections are the main focus of your peer review. The section should describe the findings of the searches, included studies, assessment of the studies and the analysis | **Peer Reviewer 1**: This review intended to respond 3 objectives:  1. What is the extent and quality of MFP intervention research? 2. What are the effects on financial outcomes of financial services embedded within healthcare settings? 3. What are the effects on health-related outcomes of financial services embedded within healthcare settings?  Although well executed, the restricted protocol/methodology did not enable authors to respond these questions. Results of this review included 4 papers only. Although I understand the need for rigorous methodologies, studies with the chosen topic of MFPs are less likely to be RCTs or have a control group, as seen in Table 3. Being more flexible with the methodology chosen could have impacted differently the results and conclusion of this review.  Because four studies were not sufficient to draw conclusions on the efficacy of MFPs, authors ended up using this review to describe the papers. Although interesting to have one compilation of studies, this review is repetitive and long. Perhaps if including studies with other methodologies and off-site interventions would substantiate different results and conclusions.  Also, because of the restricted inclusion criteria, authors used papers with a restricted length of treatment (4-6 months, 3 months, unspecified). This likely affected one of the objectives re: financial outcome. To illustrate it, two papers used tax preparation as their intervention, which is likely to help pay for specific expenses (as they come in one lump sum), but unlikely to bring financial stability/improved health. Similarly, financial counselling delivered twice may not be sufficient to change financial behavior to affect a financial outcome. The only paper that could be considered a proper intervention is Schickedanz (2023). Financial coaching monthly for 6 months is likely to impact a financial outcome, and health outcome. As described in Table 2, “Authors reported that the increase in monthly income and average savings by 6 months was greater for participants than the control group, although not statistically significant”. I would add that, qualitative studies would provide insights to the positive/negative aspects of financial interventions if included in this review.  **Peer Reviewer 2**:  **Methods**: |  | We agree with Reviewer #1 that additional studies may have been helpful, and qualitative reviews are needed. However, we maintain that a review that only includes studies with high rigor is important to answering questions about effectiveness of interventions. Including more studies with low internal validity would not have helped us in answering the question about effectiveness more definitively.  Our inclusion criteria did not restrict the length of treatment for interventions, and none of our inclusion criteria would have limited our inclusion of studies’ length of treatment, so it’s not clear what the reviewers meant by that comment.  We have added the following on page 23, “Collectively, the four studies lacked evidence for longer term financial effects (longer than six months), a variety of doses (beyond two visits), and varying length of treatment (length for 50% of the studies was unspecified)”.  We also revised the Implications for Future Research section to highlight some of these important gaps in research of these interventions**.** |
| **DISCUSSION** The discussion should explain the findings in the context of the objective of the review | **Peer Reviewer 1**: The discussion summarized the studies, and determined that the four studies did not provide an effect of MFPs on financial outcomes. Also that these studies lacked the rigor to be generalizable related to the type of settings, intervention components, geography, and household income. I am unsure that these conclusions can be drawn with 4 studies, drawn from a restricted inclusion criteria.  As the US moves into screening and intervening for social determinants of health – using the 1115 waiver – financial outcomes will be more studied, and perhaps studied more rigorously. Still early to draw conclusions about MFPs.    **Peer Reviewer 2**:  **Methods**: |  | Although early in the development of the MFP intervention, our conclusions were drawn from the evidence available. We were able to point out many aspects that could be helpful to build evidence for the future, such as standardization of outcomes, measurement of outcomes, and timing of outcomes. |
| **AUTHORS’ CONCLUSIONS** The authors’ conclusions and implications for practice and research should be consistent with the results of the review | **Peer Reviewer 1**: Authors use four articles to conclude that there is lack of evidence about whether MFPs are meeting their goals. I would say that the restrictive protocol did not allow authors to collect enough evidence to draw a better conclusion.  **Peer Reviewer 2**:  **Methods**: |  | See earlier comments about the importance of using studies with high rigor. Adding lower quality studies (single group pre-post test studies for example with low internal validity) may have increased the number of studies included, but would not have allowed us to draw any better or more certain conclusions as it relates to the effects of MFP interventions. |
| **ADDITIONAL COMMENTS ON THE REVIEW IN GENERAL** Are there other issues that should be considered in the conduct of this review? | **Peer Reviewer 1**: The text is long and truncated with so many sub-items. A more fluid text would greatly impact readability. For example, many sub-items are five sentences long. And some sub-items like “sensitive analysis”, “treatment of qualitative research”, “summary of findings and assessment of the certainty of the evidence” contain only one sentence.  **Peer Reviewer 2**:  **Methods**: |  | We can certainly see the reviewers point here; however, the sections the reviewer is referring to are part of the Campbell template and we completed all sections as required by Campbell. |

| 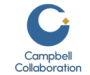 | Information Retrieval Methods Group Systematic Review Protocol Peer Review Checklist |
| --- | --- |

**Review Title:** _Medical-financial partnerships for improving financial and health outcome for lower-income Americans: A systematic review____

Instructions: This checklist is designed to aid you in organizing the evaluation of the information retrieval activities for a review protocol and to make explicit the criteria to be used during the evaluation. Each section of the checklist requires the evaluation of a specific activity. For each question in the checklist, you Did make the following assessments in the Score column:

1. Was the checklist question or criterion addressed in the **PROTOCOL**?

0: Not applicable

1: Not at all

2: Incomplete (see comments)

3: Satisfactory

Source Selection

Bibliographic Databases

Score

| **1. Were the appropriate subject databases searched?** | **3** |
| --- | --- |
| Reviewer Comments: | |

| **2. Were databases for related fields consulted?** | **3** |
| --- | --- |

Reviewer Comments:

| **3. Did the authors consult the list of databases in the Campbell Searching for Studies Guide (Kugley et al., 2016)?** | **3** |
| --- | --- |
| Reviewer Comments: | |

Grey Literature

| **4. Did the authors search for conference proceedings separately?** | **1** |
| --- | --- |
| Reviewer Comments: | |

Author comments: On page 27, we state, “In a deviation from the protocol, we did not search Conference Proceedings and Citations Index since we did not have institutional access; however, Google Scholar includes conference proceedings”. On page 1, we state that we searched for studies in Google and Google Scholar. Therefore, we searched in Google Scholar for conference proceedings.

| **5. Did the authors search for government documents separately?** | **3** |
| --- | --- |
| Reviewer Comments: | |

| **6. Did the authors search for dissertations separately?** | **3** |
| --- | --- |
| Reviewer Comments: | |

| **7. Did the authors consult the list of websites in the Campbell Searching for Studies Guide (Kugley et al., 2016)?** | **3** |
| --- | --- |
| Reviewer Comments: | |

Supplementary Searches

| **8. Did the authors conduct a hand search of journals relevant to the topic?** | **1** |
| --- | --- |
| Reviewer Comments: May not be appropriate for the topic | |

Author’s comments: A hand search of journals was not relevant to this topic. This topic did not appear in any regularity within any specific journals.

| **10. Do the authors describe how they used Google Scholar or other freely available scholarly search engines?** | **2** |
| --- | --- |

Reviewer Comments: It is unclear whether initial search included Google Scholar or if it was used only for forward citation searches.

Author comments: On page 1, we state that we searched in Google and Google Scholar for studies. On page 13, we state, “Following the procedures outlined in the protocol, we searched for and retrieved studies through a comprehensive search that included Google, Google Scholar, and 10 Electronic databases (Platform)”. We later describe that we also used Google Scholar for forward citation searching.

| **11. Did the authors consult the reference lists of reviews and/or previous studies?** | **3** |
| --- | --- |
| Reviewer Comments: | |

| **12. Did the authors contact experts in the field?** | **2** |
| --- | --- |

| **12a. If so, do the authors provide a strategy for consulting experts?** | **1** |
| --- | --- |
| Reviewer Comments: Contacted first authors of included studies | |

Author comments: We contacted first authors of included studies for data, and also asked them if they knew of other related studies that we should examine for possible inclusion. On page 2, we state, “Lastly, we contacted the first authors of the four included studies and requested information about unpublished studies, studies in progress, and published studies potentially missed in the other search activities.” We were unable to identify any other “experts” in the field.

| **15. Were non-English studies be included?** | **0** |
| --- | --- |
| **15a.** : **If so, are non-English sources included in the search?** | **0** |
| **15b. If not, did the authors provide their rationale for not doing so?** | **3** |
| Reviewer Comments: | |

Author comments: No comments required

Search Strategy

Bibliographic Databases

| **1. Are the full line-by-line search strategies included?** | **2** |
| --- | --- |
| Reviewer Comments: elements (e.g., field codes) largely missing | |

Author’s comments: In the revised Appendix A, we have added the missing field codes.

| **2. Do the authors state the platform in which the database was searched?** | **3** |
| --- | --- |
| Reviewer Comments: | |

| **3. Do the electronic searches appear sufficiently comprehensive for the topic?** | **2** |
| --- | --- |
| Reviewer Comments: See comment, below | |

Author’s comments: We reply below to the General Comments.

| **4. Does the search strategy use Boolean (AND/OR/NOT) and proximity operators appropriately?** | **3** |
| --- | --- |
| Reviewer Comments: | |

| **5. Does the search strategy include subject headings or descriptors appropriate for the topic and the database?** | **2** |
| --- | --- |
| Reviewer Comments: See comment | |

Author’s comments: We reply below to the General Comments.

**^6. Does the search strategy include keywords appropriate for the topic and the databases?^ 3**

| **6b. Is truncation and phrasing (i.e. quotation marks) used appropriately?** | **1** |
| --- | --- |
| Reviewer Comments: Truncation missing in databases where lemmatization isn't available, e.g., (Ovid) APA PsycInfo, EBSCO databases) and where it would have improved sensitivity (e.g., clinic*; stress*; toxicit*) | |

Author’s comments: We agree with the reviewer that truncation was missing in databases mentioned in the comment. However, we feel that our search strategy was thorough in other aspects, and we are confident that we did not miss any eligible studies available through our database and non-database search methods.

| **7. Is the syntax used appropriate for the database platform?** | **3** |
| --- | --- |
| Reviewer Comments: Scopus strategy omits the field codes for the first segment of the search, presumably TITLE-ABS-KEY | |

Author’s Comments: We have added the missing field codes for Scopus (TITLE-ABS-KEY)

| **8. Are line numbers combined appropriately?** | **2** |
| --- | --- |
| Reviewer Comments: Appears they used an "all in 1" approach throughout rather than combining sets | |

Author’s comments: In a note for Appendix A, we have clarified that all searches were conducted “all at once”, rather than combining sets.

| **9. Are limits or filters used (such as date or publication type limits)?** | **1** |
| --- | --- |
| **9b. If so, is appropriate justification given for the use of limits and filters?** |  |
| Reviewer Comments: Not used even where available, which may have increased precision, e.g., in CINAHL and APA PsycInfo it could have been used to limit to research studies. A Not Not on location codes in APA PsycInfo could have reduced the number of non-US based studies without losing studies not indexed with a location code. | |

Author’s comments: We used filters in each database as described in the Filters column of our table. We may have been able to use others as suggested by the reviewer; however, the limits and filters we used are provided in our search strategy for each database.

Other considerations

| **1. Was the information retrieval plan for this review sufficiently informed by the use of an information specialist?** | **3** |
| --- | --- |
| Reviewer Comments: Librarian included in Acknowledgments | |

General comments:

| Appendix A needs editing. Presumably .mp. was the field code search for (Ovid) APA PsycInfo, but that needs to be included. Similarly, one infers "All fields" was searched in the EBSCO databases, but it needs to be explicitly reported. The Scopus strategy is missing the field code for the initial facet.  Truncation is missing throughout for databases where lemmatization is not available. Quote marks are included needlessly around single words.  Strategy simply converts the PubMed strategy to the other platforms' syntax, rather than customization to each database, including use of subject headings unique to those databases with a robust thesaurus, i.e., CINAHL and APA PsycInfo. It is likely that with this topic the results will not have changed, but that is a risk not appropriate to evidence synthesis. Authors' response to protocol peer review comments regarding subject heading inclusion is based purely on definitions, not testing, which is best (standard) practice. |
| --- |

**Author’s comments:** We appreciate the close attention to detail, and the opportunity to improve Appendix A. We have added the .mp field code search for Ovid (APA PsychInfo). For the EBSCO databases, we have added “titles, abstract, subject terms, keywords” to the search terms column. For Scopus strategy, we have added “AND TITLE-ABS-KEY” to the initial facet. We acknowledge that truncation is missing in some of the databases where lemmatization is not available. We removed quote marks around single words, as they do not make a difference in searching. The edits we have made to Appendix A reflect the unique search strategy for each database.
