# Supplementary material for: Medical‐financial partnerships for improving financial and medical outcomes for lower‐income Americans: A systematic review
Source: Campbell Syst Rev. 2024 Dec 6;20(4):e70008. doi: 10.1002/cl2.70008 (PMC11621975; doi:10.1002/cl2.70008)
Supplement: Supplementary file 3 — Supporting information. [file CL2-20-e70008-s002.pdf]

Appendix A: Search Information Table

Database	Dates of Coverage	Search Fields	Filters Applied	Search Terms	Date of Search	Number of Hits
**ABI/INFORM (ProQuest)**	1900-present	Advanced: Anywhere except for no full text	English<br>All types of manuscripts	(“financial toxicity” OR “financial stress” OR “financial strain” OR “financial capability” OR “financial services” OR “economic stress” OR “financial coaching” OR “financial counseling” OR “financial mentoring” OR “financial education” OR “financial services” OR “credit counsel*” OR “tax preparation” OR “benefit screen” OR “tax filing assistance” OR “VITA site” OR VITA OR CTC OR “connection to benefit*” OR “free tax clinic” OR “earned income tax credit” OR “EITC” OR “child tax credit” OR “child savings accounts” OR “child development account*” OR “credit counseling” OR “employment assistance” OR “economic services” OR “reduce debt” OR “lower debt” OR “build credit” OR “increase credit” OR “build assets” OR “generate wealth” OR “grow income” OR “FAFSA form assistance” OR “pre-K enrollment assistance” OR “medical bill arbitration” OR “tax-time saving intervention” OR “matched savings accounts” OR “job assistance” OR “resume building” OR “savings class*”)<br><br>AND (“health clinic” OR “primary clinic” OR “primary care clinic” OR “health system” OR “clinic-based” OR “hospital” OR “clinical” OR “federally-qualified health center” OR pediatric OR	10/01/23	1,373

				“pediatric medical home” OR “medical home-embedded” OR “clinical-community partnership” OR “Medical-financial partnership” OR “clinic based financial services” OR “antipoverty medicine”)<br><br>AND (evaluation OR intervention OR treatment OR outcome OR program OR trial OR experiment OR “control group” OR “controlled trial” OR "quasi-experiment” OR random* OR empirical OR research)		
**APA PsycINFO (OVID)**	1967-Present	Advanced Search<br><br>Deselect map term to subject heading<br><br>Title<br><br>Abstract<br><br>Heading word<br><br>Table of contents<br><br>Key concepts<br><br>Original title<br><br>Tests & measures,<br><br>Mesh words	English<br><br>Human<br><br>Exclude Wire Feeds, Blogs, Podcasts, Websites, Magazines, Newspapers	("financial toxicity" OR "financial stress" OR "financial strain" OR "financial capability" OR "financial services" OR "economic stress" OR "financial coaching" OR "financial counseling" OR "financial mentoring" OR "financial education" OR "financial services" OR "credit counsel" OR "tax preparation") OR<br><br>("benefit screen" OR "tax filing assistance" OR "VITA site" OR VITA OR CTC OR "connection to benefit" OR "free tax clinic" OR "earned income tax credit" OR EITC OR "child tax credit" OR "child savings accounts" OR "child development account" OR "credit counseling" OR "employment assistance" OR "economic services" OR "reduce debt" OR "lower debt" OR "build credit" OR "increase credit" OR "build assets" OR "generate wealth" OR "grow income" OR "FAFSA form assistance" OR "pre-K enrollment assistance" OR "medical bill arbitration"	09/28/23	549

				OR "tax-time saving intervention" OR "matched savings accounts" OR "job assistance" OR "resume building" OR "savings class").mp. [mp=title, abstract, heading word, table of contents, key concepts, original title, tests & measures, mesh word]<br><br>AND ("health clinic" OR "primary clinic" OR "primary care clinic" OR "health system" OR "clinic-based" OR "hospital" OR "clinical" OR "federally-qualified health center" OR pediatric OR "pediatric medical home" OR "medical home-embedded" OR "clinical-community partnership" OR "Medical-financial partnership" OR "clinic based financial services" OR "antipoverty medicine").mp. [mp=title, abstract, heading word, table of contents, key concepts, original title, tests & measures, mesh word]<br><br>AND (evaluation OR intervention OR treatment OR outcome OR program OR trial OR experiment OR "control group" OR "controlled trial" OR "quasi-experiment" OR random* OR empirical OR research).mp. [mp=title, abstract, heading word, table of contents, key concepts, original title, tests & measures, mesh word] English.lg.<br>1 and 2 and 3 and 4		
**ProQuest Dissertations and Theses Global**	1700-present	Anywhere except full text	English<br><br>All types of manuscripts	“financial toxicity” OR “financial stress” OR “financial strain” OR “financial capability” OR	09/15/23	5,203

				"financial services" OR "economic stress" OR "financial coaching" OR "financial counseling" OR "financial mentoring" OR "financial education" OR "financial services" OR "credit counsel*" OR "tax preparation" OR<br><br>"benefit screen" OR "tax filing assistance" OR "VITA site" OR VITA OR CTC OR "connection to benefit*" OR "free tax clinic" OR "earned income tax credit" OR EITC OR "child tax credit" OR "child savings accounts" OR "child development account*" OR "credit counseling" OR "employment assistance" OR "economic services" OR "reduce debt" OR "lower debt" OR "build credit" OR "increase credit" OR "build assets" OR "generate wealth" OR "grow income" OR "FAFSA form assistance" OR "pre-K enrollment assistance" OR "medical bill arbitration" OR "tax-time saving intervention" OR "matched savings accounts" OR "job assistance" OR "resume building" OR "savings class*")<br><br>AND ("health clinic" OR "primary clinic" OR "primary care clinic" OR "health system" OR "clinic-based" OR hospital OR clinical OR "federally-qualified health center" OR pediatric OR "pediatric medical home" OR "medical home-embedded" OR "clinical-community partnership" OR "Medical-financial partnership" OR "clinic based		

				financial services" OR "antipoverty medicine")<br><br>AND (evaluation OR intervention OR treatment OR outcome OR program OR trial OR experiment OR "control group" OR "controlled trial" OR "quasi-experiment" OR random* OR empirical OR research)		
**PubMed**	1809 to Present	Advanced Search Builder<br><br>Text Availability: Abstract	Article type: All except books and documents, meta-analysis, review, systematic review, autobiography, bibliography, clinical trial veterinary, comment, data set, dictionary, directory, editorial, festschift, legal care, legislation, letter, news, newspaper article, observational study veterinary, patient education handout, personal narrative, portrait, practice guideline, video audio media, and webcast<br><br>Species: Human	((evaluation OR intervention OR treatment OR outcome OR program OR trial OR experiment OR "control group" OR "controlled trial" OR "quasi-experiment" OR random* OR empirical OR research) AND (((("Financial Stress"[Mesh:NoExp]) OR ("financial toxicity" OR "financial stress" OR "financial strain" OR "financial capability" OR "financial services" OR "economic stress" OR "economic services")) OR ("financial coaching" OR "financial counseling" OR "financial mentoring" OR "financial education" OR "financial services" OR "benefit screen" OR "tax filing assistance" OR "VITA site" OR VITA OR "Connection to benefit" OR "free tax clinic" OR "earned income tax credit" OR EITC OR "child tax credit" OR CTC OR "child savings account" OR "child development accounts" OR "credit counseling" OR "credit counsel" OR "employment assistance" OR "FAFSA form assistance" OR "pre-K enrollment assistance" OR "medical bill arbitration" OR "tax-time saving intervention" OR "matched savings accounts" OR "job assistance" OR "resume building" OR	9/18/23	63,680

			Article language: English	"savings class")) OR ("reduce debt" OR "lower debt" OR "build credit" OR "increase credit" OR "build assets" OR "generate wealth" OR "grow income"))) AND (("health clinic" OR "primary clinic" OR "primary care clinic" OR "health system" OR "clinic-based" OR "hospital" OR clinical OR "federally-qualified health center" OR pediatric OR "pediatric medical home" OR "medical home-embedded" OR "clinical-community partnership") OR ("Medical-financial partnership" OR "clinic based financial services" OR "antipoverty medicine")) Filters: Address, Case Reports, Classical Article, Clinical Conference, Clinical Study, Clinical Trial, Clinical Trial Protocol, Clinical Trial, Phase I, Clinical Trial, Phase II, Clinical Trial, Phase III, Clinical Trial, Phase IV, Comparative Study, Congress, Consensus Development Conference, Consensus Development Conference, NIH, Controlled Clinical Trial, Corrected and Republished Article, Duplicate Publication, Electronic Supplementary Materials, English Abstract, Evaluation Study, Government Publication, Guideline, Historical Article, Interview, Introductory Journal Article, Lecture, Multicenter Study, Observational Study, Overall, Periodical Index, Pragmatic Clinical Trial, Preprint, Published Erratum, Randomized Controlled Trial, Research Support, American Recovery and Reinvestment Act, Research Support, N.I.H.,		

				Extramural, Research Support, N.I.H., Intramural, Research Support, Non-U.S. Gov't, Research Support, U.S. Gov't, Non-P.H.S., Research Support, U.S. Gov't, P.H.S., Research Support, U.S. Gov't, Retracted Publication, Retraction of Publication, Technical Report, Twin Study, Validation Study, Humans, English		
**SCOPUS (Elsevier)**	1823 to Present	Article title, abstract keywords	English<br><br>Subject area: Social Sciences Economics, etc. Business, Management, etc. Medicine (not Computer science)	AND TITLE-ABS-KEY ({financial toxicity} OR {financial stress} OR{financial strain} OR {financial capability} OR {financial services} OR {economic stress} OR {economic services} OR {financial coaching} OR {financial counseling} OR {financial mentoring} OR {financial education} OR {financial services} OR {credit counsel*} OR {benefit screen} OR {tax filing assistance} OR {tax preparation} OR {VITA site} OR VITA OR CTC OR {connection to benefit} OR {free tax clinic} OR {earned income tax credit} OR eitc OR {child tax credit} OR {child savings account*} OR {child development account*} OR {credit counseling} OR {employment assistance} OR {economic services} OR {reduce debt} OR {lower debt} OR {build credit} OR {increase credit} OR {build assets} OR {generate wealth} OR {grow income} OR {FAFSA form assistance} OR {Pre-K enrollment assistance} OR {Medical bill arbitration} OR {tax-time saving intervention} OR {matched savings account} OR {job assistance} OR {resume building} OR {savings class}	09/27/23	6,907

				AND TITLE-ABS-KEY ({health clinic} OR {primary clinic} OR {primary care clinic} OR {health system} OR {clinic based} OR {clinic based} OR hospital OR clinical OR {federally qualified health center} OR {federally qualified health center} OR pediatric OR {pediatric medical home} OR (medical AND home-embedded) OR {clinical community partnership} OR {clinical community partnership} OR {anti poverty medicine} OR {anti-poverty medicine})<br><br>AND TITLE-ABS-KEY (evaluation OR intervention OR treatment OR outcome OR program OR trial OR experiment OR {control group} OR {controlled trial} OR {quasi experiment} OR {quasi experiment} OR random OR empirical OR research)		
**Web of Science Core Collection**	1990 to Present	Abstract<br><br>TS (Title, Abstract, Author Key Words, Key Words+)	English	(((TS=((“financial toxicity” OR “financial stress” OR “financial strain” OR “financial capability” OR “financial services” OR “economic stress” OR “financial coaching” OR “financial counseling” OR “financial mentoring” OR “financial education” OR “financial services” OR “credit counsel” OR “tax preparation” OR “benefit screen” OR “tax filing assistance” OR “VITA site” OR VITA OR CTC OR “connection to benefit” OR “free tax clinic” OR “earned income tax credit” OR EITC OR “child tax credit” OR “child savings accounts” OR “child development account”	09/18/23	3,484

				OR “credit counseling” OR “employment assistance” OR “economic services” OR “reduce debt” OR “lower debt” OR “build credit” OR “increase credit” OR “build assets” OR “generate wealth” OR “grow income” OR “FAFSA form assistance” OR “pre-K enrollment assistance” OR “medical bill arbitration” OR “tax-time saving intervention” OR “matched savings accounts” OR “job assistance” OR “resume building” OR “savings class”))) AND TS=((“health clinic” OR “primary clinic” OR “primary care clinic” OR “health system” OR “clinic-based” OR hospital OR clinical OR “federally-qualified health center” OR pediatric OR “pediatric medical home” OR “medical home-embedded” OR “clinical-community partnership” OR “Medical-financial partnership” OR “clinic based financial services” OR “antipoverty medicine”))) AND TS=((evaluation OR intervention OR treatment OR outcome OR program OR trial OR experiment OR “control group” OR “controlled trial” OR "quasi-experiment” OR random* OR empirical OR research)) AND LA=(English)		
**EBSCO:** Academic Search Complete<br><br>Business Source Premier<br><br>CINAHL Plus with Full Text	1887-present<br><br>1965-present	Advanced Search<br><br>Search options: titles, abstract, subject terms, keywords	English	“financial toxicity” OR “financial stress” OR “financial strain” OR “financial capability” OR “financial services” OR “economic stress” OR “Economic services” OR “financial coaching” OR “financial counseling” OR “financial mentoring” OR	10/01/23	Academic search complete: 9,279<br><br>Business Source Premier: 257

Econ Lit	1937-present<br><br>1886-present			"financial education" OR "financial services" OR "credit counsel*"<br><br>OR "tax preparation" OR "benefit screen" OR "tax filing assistance" OR "VITA site" OR VITA OR CTC OR "connection to benefit*" OR "free tax clinic" OR "earned income tax credit" OR "EITC" OR "child tax credit" OR "child savings accounts" OR "child development account*" OR "credit counseling" OR "employment assistance" OR "economic services" OR "reduce debt" OR "lower debt" OR "build credit" OR "increase credit" OR "build assets" OR "generate wealth" OR "grow income" OR "FAFSA form assistance" OR "pre-K enrollment assistance" OR "medical bill arbitration" OR "tax-time saving intervention" OR "matched savings accounts" OR "job assistance" OR "resume building" OR "savings class*")<br><br>AND ("health clinic" OR "primary clinic" OR "primary care clinic" OR "health system" OR "clinic-based" OR "hospital" OR "clinical" OR "federally-qualified health center" OR pediatric OR "pediatric medical home" OR "medical home-embedded" OR "clinical-community partnership" OR "Medical-financial) partnership" OR "clinic based financial services" OR "antipoverty medicine")<br><br>AND (evaluation OR intervention OR treatment OR		CINAHL: 1,021<br><br>Econ Lit: 23

				outcome OR program OR trial OR experiment OR “control group” OR “controlled trial” OR "quasi-experiment”OR rando m* OR empirical OR research)		

NOTE: All searches were done “all at once”, rather than combining sets.
