# Supplementary material for: Medical‐financial partnerships for improving financial and medical outcomes for lower‐income Americans: A systematic review
Source: Campbell Syst Rev. 2024 Dec 6;20(4):e70008. doi: 10.1002/cl2.70008 (PMC11621975; doi:10.1002/cl2.70008)
Supplement: Supplementary file 4 — Supporting information. [file CL2-20-e70008-s004.pdf]

Appendix B: Medical Financial Partnership Codebook

1. Report type

1. Journal article
2. Book/book chapter
3. Government report (local, state, federal)
4. Conference proceedings
5. Thesis or dissertation
6. Unpublished report (non-government, technical report) and other
7. Research brief
8. Other: (fill in)

<u>Intervention</u>

2.Intervention healthcare setting

1. Pediatric clinic (primary or specialty)
2. Federally-funded healthcare clinic (FQHC) (primary or specialty, all ages)
3. Hospital or ambulatory sites affiliated with a hospital (off main-site)
4. Primary health clinic (non-pediatric)
5. Older adult setting
6. Other: (fill in)

3.The participants receiving the services are (Check all that apply)

1. Adult/child dyads
2. Children/youth
3. Adults

4.Is financial coaching included in the intervention?

1. Yes - answer next question
2. No - skip next question

5.If yes, check all that apply <u>within</u> financial coaching

1. Financial literacy/education
2. Financial counseling
3. Credit/debt counseling
4. Free tax preparation
5. Matched college savings program
6. Employment services
7. Job training
8. One-on-one case management
9. Public benefits screening and referral
10. Community resources screening and referral
11. Assistance in applying or obtaining benefits and resources
12. Budgeting assistance
13. Expense reduction

14. Savings promotion
15. FAFSA form assistance
16. Cost-saving services
17. Other: (fill in)

6.Financial Coaching component (check all that apply)

1. Motivational interviewing
2. Goal setting
3. Other (fill in)

7.Financial service(s) provided in intervention <u>outside</u> of financial coaching (Check all that apply)

1. Financial literacy/education
2. Financial counseling
3. Credit/debt counseling
4. Free tax preparation
5. Matched college savings program
6. Employment services
7. Job training
8. One-on-one case management
9. Public benefits screening and referral
10. Community resources screening and referral
11. Assistance in applying or obtaining benefits and resources
12. Budgeting assistance
13. Expense reduction
14. Savings promotion
15. FAFSA form assistance
16. Cost-saving services
17. Other: (fill in)
18. None of the above

8.Other services provided in intervention (Check all that apply)

1. Medical bill arbitration
2. Pre-K enrollment assistance
3. Housing assistance (direct)
4. Food insecurity assistance (direct)
5. Diaper distribution (direct)
6. Other: (fill in)
7. None of the above

9.What did the control/comparison group receive?

1. Nothing or wait list
2. Treatment as usual: specify: routine clinic visits and visit reminders (text and email), referrals, standardized social needs screening
3. Specified treatment: specify
4. Other (fill in)

10.Whose financial programming was delivered?

1. Nonprofit organization
2. For-profit company
3. Government/public
4. Education (University, College, high school)
5. Other (fill in)
6. Not specified

11.Who provided the financial services? (check all that apply)

1. Researchers
2. Students (fill in: Social work)
3. Nonprofit staff
4. For-profit (financial services) staff
5. Government staff
6. Other (fill in)
7. Not specified

12.Did the staff delivering the intervention receive any training for the intervention?

1. Yes (fill in): Training from non-profit organization - go to 13
2. No - skip to 14
3. Not specified - skip to 14

13. If yes, what was the nature of the training? (check all that apply)

1. Financial coaching training
2. Motivational interviewing
3. Social determinants of health
4. Community partnerships
5. Integrated healthcare
6. Other (fill in)

14.MFP funding mechanism (check all that apply)

1. Private funding (University, grant from foundation, major donor, other philanthropy)
2. Hospital/clinic
3. Government (grants)
4. Loans/interest
5. Other: (fill in)
6. Not specified

15.Total number of sessions

1. Specified:
2. Not specified

16.Length of each session

1. Specified: 20-40 min
2. Not specified:

17. Length of treatment (# of weeks or sessions)

1. Specified: range of 4 to 6 months
2. Not specified:

18. Frequency of contact

1. Once
2. Periodically/irregular
3. Per week:
4. Per month:
5. As patient deems needed:
6. Not specified

19. Is this intervention manualized or was a specific curriculum used?

1. Yes
2. No
3. Not specified

20. Treatment format

1. Individual (one-on-one) – customized treatment of some kind
2. Group – same tx for all subjects in tx group(s)
3. Individual and group mix of standard and customized tx
4. Other
5. Not specified

<u>Methods</u>

21. Method of assignment to condition

1. Random, simple
2. Random, after matching, stratification, blocking, etc.
3. Quasi-random assigned by some naturally occurring process
4. QED with parallel cohorts
5. Not specified/not enough information to determine

22. Unit of assignment to conditions

1. Individual participant
2. Cluster: specify:
3. Dyad (parent(caregiver)/child, couple, etc)
4. Other
5. Not enough information to determine

23. If matching was used, how were groups matched?

1. Matched on pretest measure
2. Matched on demographics
3. Matched on both of the above
4. Propensity Score Matching

5. Other matching technique
6. Not enough information to determine
7. Were not matched

Results

24. Mean age of adult participants

(Give exact number or 0 for not specified)
0 = Not specified/no adult participants

25. Mean age of child/youth participants

(Give exact number or 0 for not specified)
0 = Not specified/no child/youth participants

26. Patients are

1. Primarily non-English speaking
2. Primary English speaking
3. Fairly even among English and non-English

27. % race/ethnicity

1. African American
2. Asian
3. White, non-Hispanic 49.4%
4. Hispanic/Latinx 50.6%
5. Not specified

28. Sex

1. Female % 93.8%
2. Not specified

29. Income - If author specifies income description, select (1, 2 or 3). If there is no description, select 4.

1. Low Income
2. Low and moderate income
3. All income levels
4. Income mean/median specified (fill in)
5. Not specified

30. How was fidelity assessed?

1. Not measured/not reported
2. Researcher observations
3. Interviews/focus groups of participants
4. Surveys of participants
5. Participant logs
6. Administrative records
7. Checklists

8. Other - workflow time motion analyses
9. Not specified

31. What was the results of the fidelity assessment
    1. Fidelity was acceptable
    2. Fidelity was problematic (low)
    3. Outcomes of fidelity were not reported

32.Tx sample size at assignment (give exact number)
33.Tx analytic sample size (give exact number)
34.% Attrition (dropout) (calculate analytic sample size/at assignment)

35.Control group sample size at assignment (give exact number)
36.Control group analytic sample size (give exact number)
37.% Attrition (dropout) (calculate analytic sample size/at assignment)

Outcomes Measured:

<u>38.Health - Child outcomes (Check all that apply)</u>

1. Visit adherence (Well child)
2. Vaccination schedule adherence
3. Other (fill in)
4. None

<u>39.Health - Adult outcomes</u>

1. Prevention health visit
2. Other (fill in)
3. None

<u>40.Financial</u>

1. Income
2. Savings
3. Financial knowledge
4. Financial attitude
5. Tax filing status
6. Tax refund receipt and amount
7. Debt
8. Credit scores
9. Other (fill in)

41.Results of statistical comparisons of pretest differences on outcomes
1. No statistical comparisons made

2. No statistically significant differences
3. Statistically significant differences found

42. Which outcomes did they compare at pretest? (Fill in)

43. Results of statistical comparisons of pretest differences on demographics (e.g., race, ethnicity)

1. No statistical comparisons made
2. No statistically significant differences
3. Statistically significant differences found

44. If groups were non-equivalent at baseline, were statistical controls used?

1. Yes
2. No
3. Were equivalent
4. Not enough information to determine

45. Role of the evaluator/author/research team or staff in the intervention

1. Researcher involved in designing the intervention
2. Researcher involved in delivering the intervention only
3. Researcher was involved in designing and delivering the intervention
4. Researcher was not involved in designing or delivering the intervention
5. Not specified/unsure
